# Runx1+ vascular smooth muscle cells are essential for hematopoietic stem and progenitor cell development in vivo

**DOI:** 10.1038/s41467-024-44913-z

**Published:** 2024-02-23

**Authors:** Zaniah N. Gonzalez Galofre, Alastair M. Kilpatrick, Madalena Marques, Diana Sá da Bandeira, Telma Ventura, Mario Gomez Salazar, Léa Bouilleau, Yvan Marc, Ana B. Barbosa, Fiona Rossi, Mariana Beltran, Harmen J. G. van de Werken, Wilfred F. J. van IJcken, Neil C. Henderson, Stuart J. Forbes, Mihaela Crisan

**Affiliations:** 1 Centre for Cardiovascular Science, The University of Edinburgh, Edinburgh, UK; 2 Centre for Regenerative Medicine/Institute for Regeneration and Repair, The University of Edinburgh, Edinburgh, UK; 3 Centre for Inflammation Research/Institute for Regeneration and Repair, The University of Edinburgh, Edinburgh, UK; 4 Cancer Computational Biology Center, Erasmus MC Cancer Institute, University Medical Center, 3000 CA Rotterdam, The Netherlands; 5 Department of Urology, Erasmus MC Cancer Institute, University Medical Center, 3000 CA Rotterdam, The Netherlands; 6 Department of Immunology, Erasmus MC Cancer Institute, University Medical Center, 3000 CA Rotterdam, The Netherlands; 7 Center for Biomics, Department of Cell Biology, Erasmus MC University Medical Centre, 3015 GE Rotterdam, The Netherlands; 8 MRC Human Genetics Unit, Institute of Genetics and Cancer, The University of Edinburgh, Edinburgh, UK

**Keywords:** Cell biology, Haematopoiesis, Cardiovascular biology

## Abstract

Hematopoietic stem cells (HSCs) produce all essential cellular components of the blood. Stromal cell lines supporting HSCs follow a vascular smooth muscle cell (vSMC) differentiation pathway, suggesting that some hematopoiesis-supporting cells originate from vSMC precursors. These pericyte-like precursors were recently identified in the aorta-gonad-mesonephros (AGM) region; however, their role in the hematopoietic development in vivo remains unknown. Here, we identify a subpopulation of NG2^+^Runx1^+^ perivascular cells that display a sclerotome-derived vSMC transcriptomic profile. We show that deleting Runx1 in NG2^+^ cells impairs the hematopoietic development in vivo and causes transcriptional changes in pericytes/vSMCs, endothelial cells and hematopoietic cells in the murine AGM. Importantly, this deletion leads also to a significant reduction of HSC reconstitution potential in the bone marrow in vivo. This defect is developmental, as NG2^+^Runx1^+^ cells were not detected in the adult bone marrow, demonstrating the existence of a specialised pericyte population in the HSC-generating niche, unique to the embryo.

## Abstract (web summary)

Hematopoietic stem cells are supported by niche cells that help balance stem cell self-renewal and differentiation. Here they show that Runx1 deletion in the embryonic perivascular HSC niche impairs hematopoietic development in vivo and causes transcriptional changes in pericytes/vSMCs, endothelial cells and hematopoietic cells in the murine AGM.

## Introduction

During the lifespan of an organism, hematopoietic stem cells (HSCs) continuously produce all the essential cellular components of the blood. Aberrant hematopoiesis leads to blood-related diseases such as leukemia and anemia. Treating these diseases requires blood transfusions or bone marrow transplants, which are limited to the availability of compatible donors. Developing a method to produce functional HSCs in a dish could increase treatment efficiency and reduce mortality. The current inability to specify HSCs in vitro highlights today’s challenge in generating these clinically important cells and the necessity to understand how they arise in vivo.

In the mouse, adult-type HSCs are first detected at developmental day (E)10.5 in the aorta-gonad-mesonephros (AGM) region, where they emerge from hemogenic endothelium^1–8^. This takes place under the regulatory influence of the surrounding microenvironment. The specific HSC inducing niche includes cell types that release and/or respond to a combination of pro-hematopoietic factors involved in this process. In addition to the sympathetic nervous system (SNS)^9^, ventral tissue^10^, immune system^11^ and neural crest cells^12^, mesenchymal stromal cells have been shown to regulate HSC emergence and/or HSC maintenance^13–17^. Some of these mesenchymal cells support HSCs in vitro better than others^18^ suggesting that cultured mesenchymal cell lines are heterogeneous^19^.

A previous study found that hematopoietic supportive mesenchymal stromal cells follow a vascular smooth muscle cell (vSMC) differentiation pathway in culture^20^. Indeed, alongside mesenchymal markers found in E11 AGM stromal cell lines, markers that identify early/intermediate vSMC lineage such as αSMA and SM-actinin have also been described^20^. These findings strongly suggest that at least some hematopoietic supportive cultured cells originate from vSMC precursors in vivo. Roostalu et al. recently identified such vSMC precursors that resemble pericytes (PC) in the AGM. These cells express NG2 and CD146 and their emergence in the AGM at E10.5 coincides with the timeframe when HSCs are first generated^21^. However, whether PC/vSMCs play a role in HSC emergence in vivo has not yet been documented.

In vivo, AGM PC/vSMCs are highly heterogeneous. Previous work showed that a subset of sub-aortic mesenchymal cells express Runx1^22–24^, a key transcription factor required for HSC generation in the mouse embryo^24–26^. It is not known whether Runx1 in PC/vSMCs plays a role in HSC development.

In this work, we hypothesised that PC/vSMCs expressing Runx1 are involved in AGM hematopoietic stem/progenitor cell (HSPC) development in vivo. Using single-cell RNA-sequencing (scRNA-seq), and 3-dimensional imaging in transgenic mice, we show a perivascular and hematopoietic functional and developmental relationship in the AGM at both cellular and molecular levels.

## Results

### A subpopulation of subaortic mesenchyme in the AGM co-expresses NG2 and Runx1

**Fig. 1:**
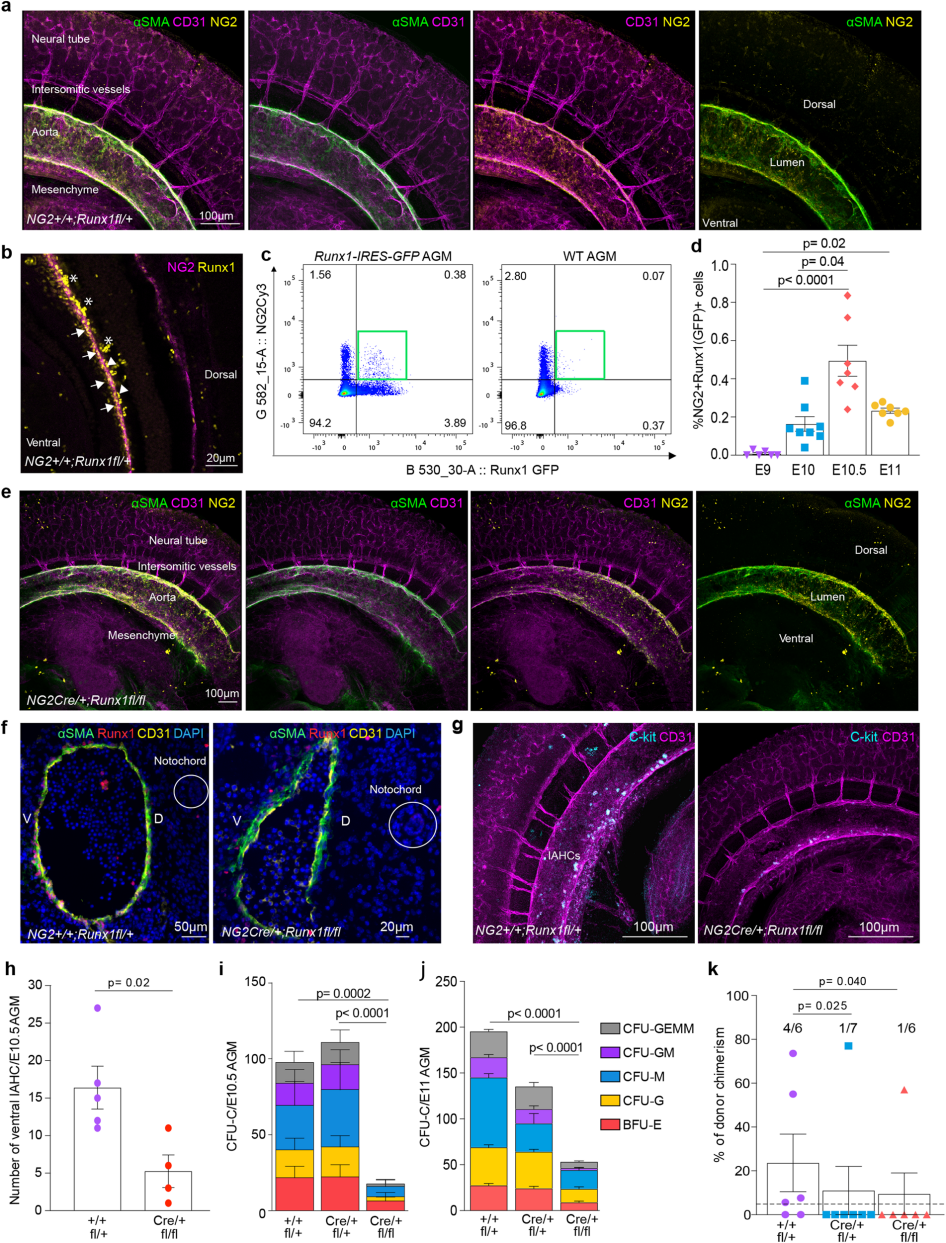
A subset of sub-aortic mesenchyme co-expresses NG2 and Runx1 and controls HSPC development in vivo. **a** Three-dimensional (3D) wholemount immunostaining with αSMA, CD31 and NG2 of E10.5 (31–38 somite pairs (sp)) WT dorsal aorta; **b** NG2 and Runx1 expression on single plane wholemount WT E10.5 sections. NG2^+^Runx1^+^vSMCs (arrows), hemogenic endothelial cells (arrowheads) and intra-aortic hematopoietic clusters (IAHCs, stars) (Table S1); **c** Representative example of flow cytometric analysis of NG2^+^Runx1(GFP)^+^ (green box) in E10.5 *Runx1-IRES-GFP* AGM and E10.5 WT control. **d** Percentages of NG2^+^Runx1(GFP)^+^ cells in E9 (21-25sp) body (*n* = 6), E10/E10.5/E11 AGMs (*n* = 8/7/7), *N* = 5, Kruskal-Wallis and Dunn’s *post-hoc* test. **e** Representative examples of wholemount 3D-images showing αSMA, CD31 and NG2 in E10.5 cKO dorsal aortae; **f** αSMA, Runx1 and CD31 immunofluorescence of E11 WT and cKO transversal frozen sections; *n* = WT/cKO: 2/2, *N* = 2. **g** cKit and CD31 wholemount 3D-images in E10.5 WT and cKO AGM; **h** Number of intra-aortic hematopoietic clusters (IAHCs) in E10.5 AGM; *n* = WT/KO: 5/4, *N* = 4. Number of colony forming unit-culture (CFU-C) in **i** E10.5 (31-38sp) AGM; *n* = WT/HET/KO: 14/10/5 embryos; *N* = 7 and **j** E11 (43–52sp) AGM; *n* = WT/HET/KO: 22/8/19 embryos; *N* = 11; one-way ANOVA and Tukey’s post-hoc test (Table S2). **k** Percentages of donor cell chimerism 4-months post-transplantation of 6 E11 WT *(NG2*^*+/+*^*;Runx1*^*fl/+*^or *NG2*^*+/+*^*;Runx1*^*fl/fl*^*)*, 7 HET (*NG2-Cre;Runx1*^*fl/+*^) and 6 cKO AGMs (*NG2-Cre;Runx1*^*fl/fl*^) into sub-lethally adult irradiated recipients (1xAGM cells transplanted/recipient; *N* = 4). Each dot represents one recipient. Mice are reconstituted when ≥5% donor cells are found in the host peripheral blood (dashed line)**;** one-tailed Z score test for two population proportions (Tables S3 and S4). For wholemount staining in **a**, **b**, **e**, **g**: WT/cKO (*N* = 6/4): αSMA (*n* = 9/7), CD31 (*n* = 10/7), cKit (*n* = 3/2), NG2 (*n* = 3/1) and WT Runx1 (*n* = 4) in 3 distinct combinations (Table S1). D = dorsal, V = ventral. N = number of independent experiments; n = number of biological samples (embryos). All data are presented as mean values ± SEM. Source data for **d**, **h**, **i**, **j** and **k** are provided as a Source Data file.

We examined the expression of PC/vSMC markers in the dorsal aorta of E10.5 and E11 mouse embryos. Wholemount immunostaining and immunohistochemistry on frozen sections were performed using PC/vSMC markers NG2 or αSMA with CD31, an endothelial and HSPC marker (Table S1). Imaging analysis showed that NG2^+^αSMA^+^CD31^-^ vSMCs surround NG2^-^αSMA^-^CD31^+^ endothelial cells (Figs. 1a, S1a, b), confirming previous reports^27^. Further to its expression in hematopoietic and hemogenic endothelial cells, Runx1 was also detected in the sub-aortic mesenchyme^22,23^. Therefore, we hypothesised that at least some of these cells also express NG2. We first confirmed that both intra-aortic hematopoietic cell clusters (IAHCs) (Fig. 1b, stars) and hemogenic endothelial cells (Fig. 1b, arrowheads) are Runx1^+^; we also identified a subpopulation of NG2^+^ PC/vSMCs, mainly located in the ventral aspect of the dorsal aorta, that also express Runx1 (Fig. 1b, arrows). Other Runx1^+^ cells in the perivascular area do not express NG2 (Fig. 1b). Finally, we confirmed our recent study^28^ that some cells around the notochord express NG2 in the trunk (Fig. S1a, circle). However, these peri-notochord cells do not express αSMA, CD31 (Fig. S1a, circle) nor Runx1 (Fig. S1e–f). To confirm the presence of NG2^+^Runx1^+^ cells in the E11 AGM, we used *Runx1-IRES-GFP* mouse embryos^29^. In these GFP knock-in mice, GFP intensity correlates with *Runx1* expression level. Flow cytometric analysis showed the presence of a distinct population of NG2^+^Runx1(GFP)^+^ cells in the AGM (Fig. 1c). These cells first appear at E10, in line with the presence of Runx1 in mesenchymal cells^30^ and importantly, their frequency peaks at E10.5 (Fig. 1d). Together, these data show that in the AGM, a subset of the sub-aortic mesenchyme expresses both NG2 and Runx1 and that the highest frequency of these cells coincides with the onset of HSC generation at E10.5.

### *Runx1* deficiency in NG2^+^cells impairs AGM hematopoiesis

*Runx1* deletion in endothelial cells impairs HSC emergence in the AGM^24–26^. However, the effect of *Runx1* deletion in PC/vSMCs on hematopoiesis in vivo is still unknown. To address this, we examined conditional knock-out (cKO) *NG2-Cre;Runx1*^*fl/fl*^ mouse embryos. In previous studies, the *NG2-Cre* mouse strain revealed a role for pericytes in supporting both fetal liver and adult bone marrow HSC maintenance^31,32^. Our data shows that E10.5 and E11 cKO embryos do not exhibit visible vascular abnormalities. This was confirmed by the normal expression of CD31, αSMA and NG2 (Figs. 1e, f, S1c, d) in the AGM. In contrast, αSMA^+^Runx1^+^ PC/vSMCs with low expression of Runx1 were reduced in the cKO dorsal aorta compared to WT littermate controls (Figs. 1f, S1e–g). CD31^+^Runx1^+^ endothelial cell number and frequency was also decreased (Fig. S1g). Furthermore, CD31^+^cKit^+^ IAHC numbers were significantly reduced by three-fold (*p* = 0.02) (Fig. 1g, h). Hematopoietic progenitor (HP) assays were performed to test if hematopoietic function was affected. All HP numbers were significantly reduced in cKO AGMs at both E10.5 (Fig. 1i, Table S2) and E11 (Fig. 1j, Table S2). To test whether definitive HSCs were also affected, we performed HSC assays in vivo. At 1- and 4-months post-transplantation of AGM cells into sub-lethally irradiated mice, chimerism and multilineage reconstitution were examined by flow cytometry in the peripheral blood. Compared to the WT littermate control group, in which 66.7% (4 out of 6) recipients were reconstituted, only 14.3% (1 in 7, *p* = 0.025) and 16.7% (1 in 6, *p* = 0.040) mice injected with heterozygous or homozygous cKO AGMs, respectively, were reconstituted over the long term (Fig. 1k, Tables S3–4). These findings indicate that the absence of *Runx1* in aortic NG2^+^ cells impairs HSC generation and/or maintenance and HP development in the AGM.

### NG2^+^Runx1^+^ cells do not contribute to hematopoietic lineages in the AGM

To test whether NG2^+^ cells contribute directly to hematopoietic lineages, we isolated NG2^+^ and NG2^+^Runx1(GFP)^+^cells from E11 WT and *Runx1-IRES-GFP* AGMs, respectively, and seeded them in methylcellulose. In parallel, NG2^-^ or NG2^-^cKit^+^ cells were sorted as controls. HPs were exclusively found in the NG2^-^ cell fractions. Neither NG2^+^ cells (Fig. S2a) nor NG2^+^Runx1(GFP)^+^cells (Fig. S2b) gave rise to hematopoietic cell colonies in vitro (Table S5). To further assess whether NG2^+^ cells are hematopoietic precursors, we crossed *NG2-Cre* mice with a knock-in reporter mouse line in which tdTomato is preceded by a transcriptional stop flanked by two loxP sites under the Rosa26 promoter. In these mice, NG2^+^ cells and their progeny are tdTomato^+^. E11 AGM-derived tdTomato^+^ and tdTomato^-^ cells were sorted and seeded in methylcellulose. HPs were only found in the tdTomato^-^ cell fraction (Fig. S2c, Table S5) reinforcing the observation that NG2^+^ cells and their progeny do not contribute to hematopoietic lineages at this stage. Flow cytometric analysis confirmed the presence of tdTomato in a subset of NG2^+^ cells in the E11 AGM (Fig. S2d), validating our mouse model, while no overlap was found between tdTomato and CD45, a hematopoietic cell marker (Fig. S2e). We next performed immunohistochemistry on *NG2-Cre;tdTomato*^*fl/+*^ frozen sections and confirmed the expression of tdTomato in a subset of αSMA^+^ cells (Fig. S2f) in the E11 AGM. CD31^+^ cells did not express tdTomato (Fig. S2g). Further analysis revealed that cells expressing hematopoietic markers F4/80 and CD45 do not co-express NG2 nor αSMA (Fig. S2h). Together, these data indicate that NG2^+^ cells do not contribute to the AGM HSPC pool and suggest that NG2^+^Runx1^+^ PC/vSMCs act as a supportive niche to maintain hematopoietic activity in the AGM.

### Deletion of *Runx1* in NG2^+^cells affects selective hematopoietic progenitor types in embryonic organs other than the AGM

In the early developing embryo, HSPCs reside in other intra-embryonic and extra-embryonic hematopoietic organs such as the head, fetal liver (FL), placenta and yolk-sac (YS). Flow cytometric analysis of these organs harvested from *Runx1-IRES-GFP* mouse embryos also confirmed the presence of NG2^+^Runx1(GFP)^+^ cells (Fig. S3a, b). We next performed in vitro HP functional assays with cells harvested from all organs and genotypes of *NG2-Cre;Runx1fl* at both E10 and E11 developmental stages. No significant differences were found when comparing the total CFU-C numbers between genotypes in most organs (Fig. S3c, d, Tables S6–7). A significant increase of total number of CFU-C was observed in E10 AGM in both heterozygous and cKO mouse embryos (Fig. S3c). When analyzed individually, a significant increase in the number of erythroid colonies was detected in the cKO compared to WT littermate (*p* = 0.0149) (Table S6). Likewise, a 2.8-fold increase in the number of erythroid colonies was detected in the E11 cKO head compared to the WT littermate (*p* = 0.01), while the total number of CFU-C in the E11 head remained unchanged (Table S7). Moreover, we found a significant decrease in both CFU-GM (*p* = 0.016) and CFU-GEMM (*p* = 0.039), between WT and cKO YS (Table S7), possibly due to the defect found in the E11 AGM.

To test whether HSC activity increases in the FL due to the possible migration of AGM HSCs, E11 FL cells from all genotypes were transplanted into sub-lethally irradiated recipient mice. Neither the donor chimerism nor the percentage of reconstituted mice by donor cells showed changes between the groups (Fig. S3e). Compared to *NG2*^*+/+*^;*Runx1*^*fl/+*^ WT littermates, in which 70% of recipients (7 out of 10) were reconstituted, mice injected with *NG2-Cre:Runx1*^*fl/+*^ heterozygous or *NG2-Cre:Runx1*^*fl/fl*^ cKO E11 FL showed similar reconstitution over the long term, with 67% (2 out of 3, *p* = 0.348) and 60% (3 out of 5, *p* = 0.421) reconstituted mice, respectively (Fig. S3e, Tables S3–4). Since the deletion of Runx1 in NG2^+^ cells only affects HSPCs in the AGM, immunohistochemistry on WT embryonic head and placenta was performed to localise NG2^+^Runx1^+^ cells. The rare NG2^+^Runx1^+^ double positive cells identified did not seem to be perivascular (Fig. S3f, stars). In line with this observation, we found that Runx1 and αSMA do not overlap when NG2 and αSMA were expressed in PC/vSMCs (Fig. S3f, arrowheads). Instead, the head contains few NG2^+^αSMA^-^ that are F4/80^+^, suggesting that NG2^+^Runx1^+^ cells are macrophages (Fig. S3f, arrowhead). Overall, our data shows that the deletion of *Runx1* in NG2^+^ cells only affects selective HSPC subsets in non-AGM hematopoietic organs in the E11 mouse embryo.

### AGM PC/vSMCs have a distinct transcriptome from endothelial and hematopoietic cells

**Fig. 2:**
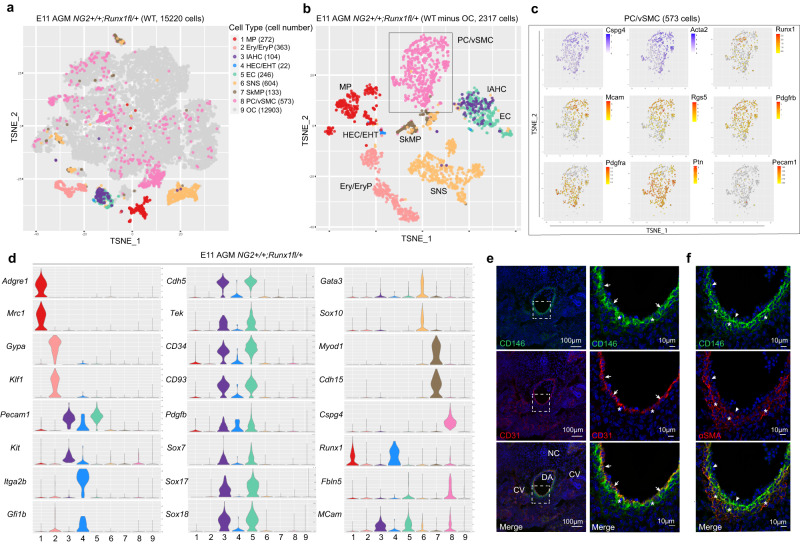
AGM PC/vSMCs have a distinct transcriptome from endothelial and hematopoietic cells. **a** t-SNE plot highlighting eight populations of interest identified in the E11 WT AGM. Each dot represents one cell and colours represent cell clusters as indicated. The number of cells in each population is shown in brackets. MP (macrophages); Ery/EryP (erythroid/progenitors); IAHC (intra-aortic hematopoietic clusters); HEC/EHT (hemogenic endothelial cells including those that enter endothelial-to-hematopoietic transition); EC (endothelial cells); SNS (sympathetic nervous system); SkMP (skeletal muscle progenitors), PC/vSMC (pericytes/vascular smooth muscle cells, *NG2*^*+*^*Acta2*^*+*^). Other cells (OC) are coloured in grey. **b** t-SNE plot highlighting the eight populations identified after excluding all other (grey) cells. **c** Zoom into PC/vSMC cluster (black rectangle) further show the presence or the absence of selected genes that characterise this population and confirms the presence of Runx1 in a subset of cells. **d** Violin plots showing distribution of expression for selected genes that contributed to the identification of cell clusters. Immunohistochemistry on frozen E11 WT sections stained with **e** CD146/CD31/DAPI and **f** CD146/αSMA/DAPI, *n* = 2 samples tested, *N* = 2 independent experiments. Arrows: vascular cells, asterisks: perivascular cells. DA: dorsal aorta, CV: cardinal veins, NC: notochord. Source data for e (first column, 20X) is provided as a Source Data file.

To better understand the role of Runx1 in the HSC-generating microenvironment, single-cell RNA-sequencing (scRNA-seq) on *NG2*^*+/+*^*;Runx1*^*fl/+*^ E11 AGM was performed. We used graph-based clustering and known marker distribution to define and investigate the gene expression profiles of various populations that reside in the E11 AGM and identified eight populations of interest (Fig. 2a, b). The co-expression of *Cspg4* (NG2) and *Acta2* (αSMA) in the PC/vSMC population was confirmed (Fig. 2c). This population is also enriched in *Rgs5, Pdgfrb* and *Pdgfra* in line with our previous work^28^_,_ and a subset of these cells express *Runx1* (Fig. 2c, d), confirming our imaging and flow cytometric analysis. The expression of *Mcam* (CD146 or S-ENDO1), a pericyte/vSMC precursor marker recently identified in a subset of NG2^+^ cells in the E11 AGM^21^ and upregulated in AGM hematopoiesis supportive stromal cell lines^19^, was detected in a subset of PC/vSMCs, partially overlapping with *Runx1*^*+*^ cells (Fig. 2c, d). However, *Mcam* was mainly enriched in endothelial cells (ECs) and also in subpopulations of hemogenic endothelial cells, including those entering endothelial-to-hematopoietic transition (HEC/EHT), IAHCs and SNS cells (Fig. 2d), confirming published work including ours^28,33^. Immunostainings with CD146 and CD31 on E11 WT AGM frozen sections further validated our sequencing analysis at the protein level: both CD31^+^ endothelial cells (Fig. 2e, f, arrows) and αSMA^+^ PC/vSMCs (Fig. 2f, stars) are CD146^+^. Importantly, *Pecam-1* (CD31) expression in PC/vSMCs was low/negative in our scRNA-seq data (Fig. 2c, d), in line with our immunohistochemistry, confocal imaging, and our recent published work^28^. Other genes expressed by hematopoietic and hemogenic/endothelial cells such as *Adgre1* (F4/80), *Mrc1* (CD206), *Cdh5* (VE CADHERIN), *Tek* (TIE-2), *CD34*, *CD93*, *Pdgfb*, *Sox7, Sox17, Sox18, Gfi1b*, and *Itga2b* (CD41) were not expressed in PC/vSMCs (Fig. 2d). These genes were used to distinguish populations of macrophages (MPs), IAHCs, HEC/EHT, and ECs (Fig. 2a–d). Erythroid cells and erythroid progenitors (Ery/EryP; *Gypa*/CD235^+^), SNS (*Gata3*^+^) and skeletal muscle progenitors (SkMP; *MyoD1*^+^, *Cdh15*^+^) were also identified (Fig. 2a–d). *Kit* was expressed in all IAHCs and in a subset of PC/vSMCs, while *Kit* expression in HEC/EHTs was low (Fig. 2d). Altogether, these data show that we successfully captured multiple cell types that comprise the E11 AGM, including a population of *Runx1*^*+*^ PC/vSMCs which constitutes 19.7% of all *NG2*^*+*^*Acta2*^*+*^ PC/vSMCs cells. Furthermore, the transcriptome of *Cspg4*^*+*^*Runx1*^*+*^ non-hematopoietic non-endothelial PC/vSMCs was found to partially overlap with that of the *Cspg4*^*+*^*Mcam*^*+*^ PC/vSMC precursors previously described^21^.

### *Cspg4*^*+*^*Runx1*^*+*^ AGM cells display a sclerotome-derived vSMC transcriptomic profile

**Fig. 3:**
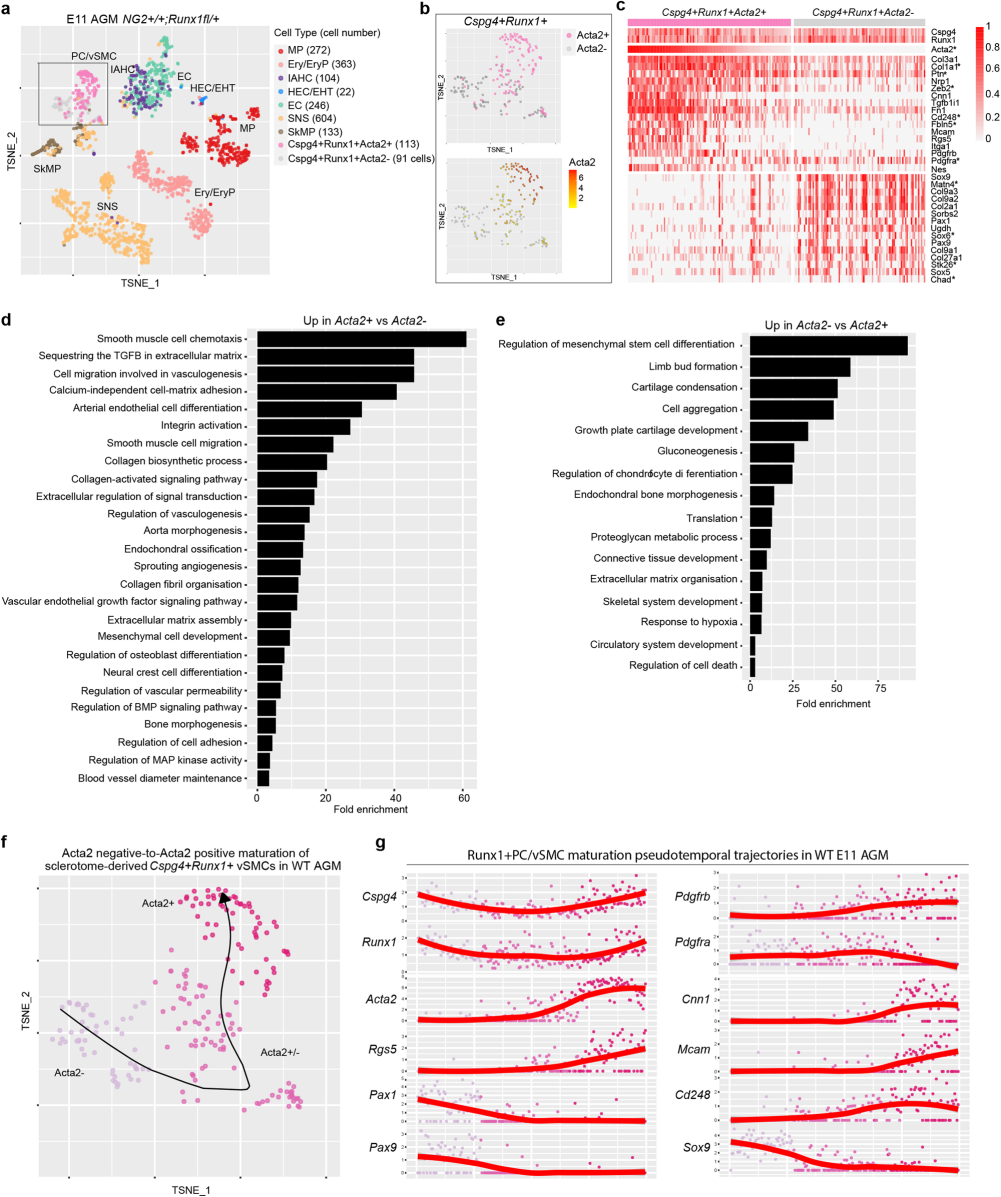
*NG2*^*+*^*Runx1*^*+*^ AGM cells display a sclerotome-derived vSMC transcriptomic profile. **a** t-SNE plots showing the distribution of *Runx1* and *Acta2* expression in *NG2*^*+*^*Runx1*^*+*^ cells in the WT E11 AGM after excluding all other (grey) cells found in the Fig. 2a. **b** Zoom into *NG2*^*+*^*Runx1*^*+*^ cluster (black rectangle) shows the presence or the absence of *Acta2*. **c** Heatmap showing the expression of *Cspg4* and *Runx1* and 15 selected genes out of 25 top significantly upregulated genes in WT *NG2*^*+*^*Runx1*^*+*^*Acta2*^*+*^ cells (upper half) and *NG2*^*+*^*Runx1*^*+*^*Acta2*^*-*^ cells (bottom half) at single cell level; *Runx1 potential target genes. *Pdgfra* and *Ptn* genes were next added to inform their expression in both populations. Barplot of fold enrichment for selected GO biological processes significantly overrepresented in genes significantly upregulated in both **d** WT *NG2*^*+*^*Runx1*^*+*^*Acta2*^*+*^ and **e** *NG2*^*+*^*Runx1*^*+*^*Acta2*^*-*^ cells. **f** t-SNE of WT E11 AGM cells, overlaid with principal pseudotime curve inferred by Slingshot, predicting a lineage from *NG2*^*+*^*Runx1*^*+*^*Acta2*^*-*^ cells to *NG2*^*+*^*Runx1*^*+*^*Acta2*^*+*^ cells. **g** WT *NG2*^*+*^*Runx1*^*+*^cells arranged in pseudotime (x-axis) based on the inferred curve. Y-axis represents log normalised gene expression.

Our scRNA-seq analysis revealed that not all *Cspg4*^*+*^*Runx1*^*+*^ cells in the E11 AGM express *Acta2* (Fig. 3a, b). We therefore investigated if *Cspg4*^*+*^*Runx1*^*+*^*Acta2*^*−*^ cells are PC/vSMCs which had not yet acquired *Acta2* expression. Differential expression analysis of *Acta2*^*+*^ versus *Acta2*^*−*^ cells within the *NG2*^*+*^*Runx1*^*+*^ cell population in the WT AGM revealed that markers of sclerotome-derived vSMCs such as *Sox9, Pax1*, *Pax9* and *Col2a1*^34^ are among the highest upregulated genes in *Cspg4*^*+*^*Runx1*^*+*^*Acta2*^*−*^ cells (Fig. 3c). In contrast, *Cspg4*^*+*^*Runx1*^*+*^*Acta2*^*+*^ cells are enriched in genes that identify more mature pericytes such as *Acta2, CD248, Mcam, Rgs5* or *Pdgfrb* (Fig. 3c), some of which are potential *Runx1* target genes (star). *Pdgfra* and *Ptn* genes were recently associated with Runx1^+^ subaortic (non-smooth muscle) mesenchymal cells with possible role in hematopoiesis in the E10.5 AGM of the mouse embryo^35^. Our scRNA-seq analysis show that, in E11 AGM, *Pdgfra* and *Ptn* are also expressed in *Cspg4*^*+*^*Runx1*^*+*^ cells with no significant difference between *Acta2*^*+*^ and *Acta2*^*−*^ (Fig. 3c). Further analysis showed that Gene Ontology (GO) biological processes significantly enriched in *Cspg4*^*+*^*Runx1*^*+*^*Acta2*^*+*^ cells include smooth muscle cell chemotaxis and migration, collagen-activated signalling pathway, neural crest cell differentiation and regulation of BMP signalling (Fig. 3d), previously shown by our laboratory to control in vivo HSPC generation in the mouse AGM^28^. In *Cspg4*^*+*^*Runx1*^*+*^*Acta2*^*−*^ cells, significantly enriched GO biological processes include mesenchymal stem cell differentiation and cartilage and bone development (Fig. 3e), consistent with the sclerotome origin of these cells.

Indeed, PC/vSMCs in the AGM have been shown to originate from the sclerotome and display markers of this compartment at least during the early phases of mural cell recruitment^36^. A recent study showed that the maturation of sclerotome-derived vSMCs in the mouse AGM depends on a transcriptional switch from a sclerotome signature with the repression of *Pax1, Scx* and *Sox9*, and activation of *Acta2* and other vSMC genes^34^.

To test whether *NG2*^*+*^*Runx1*^*+*^*Acta2*^*−*^ cells follow a maturation trajectory towards *Cspg4*^*+*^*Runx1*^*+*^*Acta2*^*+*^ vSMCs, we performed cell lineage inference with Slingshot, a trajectory inference method for scRNA-seq data that can incorporate knowledge of developmental markers. Having defined a cluster of *Cspg4*^*+*^*Runx1*^*+*^*Acta2*^*−*^ cells as an origin, Slingshot infers a cell lineage and constructs a pseudotime curve representing that lineage (Fig. 3f, arrow). Gene expression along pseudotime shows that sclerotome markers such as *Sox9, Pax1* and *Pax9* are gradually downregulated while markers of mural cells such as *Acta2, Rgs5, Pdgfrβ, Cnn1, Mcam and CD248*, are gradually upregulated in an inferred transition from *Cspg4*^*+*^*Runx1*^*+*^*Acta2*^*−*^ to *Cspg4*^*+*^*Runx1*^*+*^*Acta2*^*+*^ cells (Fig. 3g). Our scRNA-seq analysis shows that *Cspg4*^*+*^*Runx1*^*+*^ AGM cells display a sclerotome-derived vSMC transcriptomic profile.

### The genetic programme of PC/vSMCs is altered in *NG2-Cre;Runx1*^*fl/fl*^ cKO E11 AGM

**Fig. 4:**
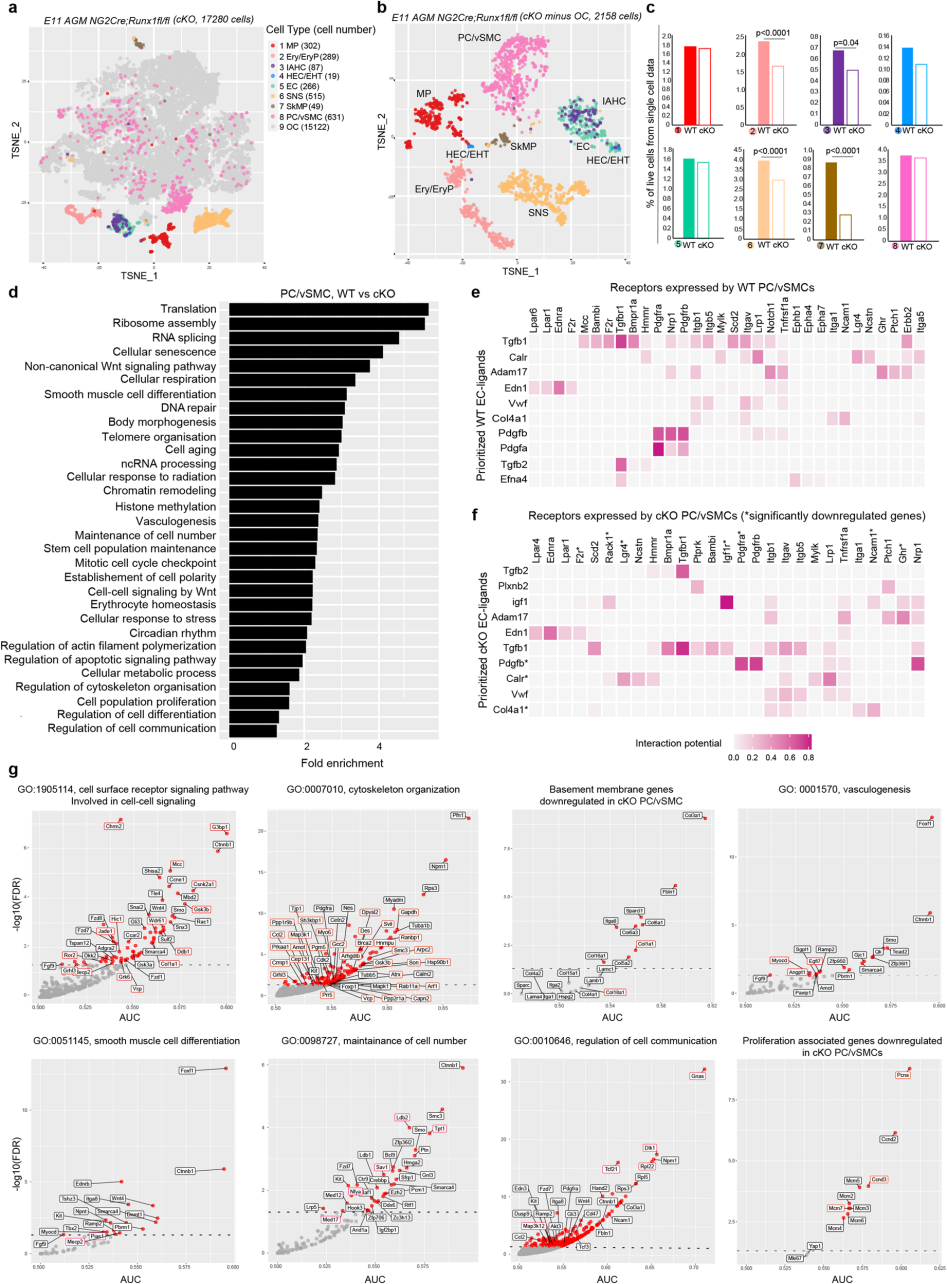
The genetic programme of PC/vSMCs is altered in *NG2-Cre;Runx1*^*fl/fl*^ cKO E11 AGM. **a** t-SNE plot showing eight populations of interest found in the E11 cKO AGM. Each dot represents one cell and colours represent cell clusters as indicated. MP (macrophages); Ery/EryP (erythroid/progenitors); IAHC (intra-aortic hematopoietic clusters); HEC/EHT (hemogenic endothelial cells including those that enter endothelial-to-hematopoietic transition), EC (endothelial cells); SNS (sympathetic nervous system); SkMP (skeletal muscle progenitors); PC/vSMC (pericytes/vascular smooth muscle cells, *NG2*^*+*^*Acta2*^*+*^*)*. Other cells (OC) are coloured in grey. The number of cells in each cluster is shown in brackets. **b** t-SNE plot highlighting the eight populations identified after excluding all other (grey) cells. **c** Percentage of single live cells found in each E11 AGM sample (cell number/total cells) defined by scRNA-seq in WT (full bars) and cKO (empty bars) AGMs. Colours and numbers correspond to each population defined in **a**; chi-squared two-tailed test was used for comparison. **d** Barplot of fold enrichment for selected GO biological processes significantly overrepresented in genes significantly downregulated in cKO PC/vSMCs compared to their WT counterparts. Heatmap of ligand-receptor interactions inferred by NicheNet from **e** WT and **f** cKO E11 AGM cells. Colour represents the interaction potential score between the 10 top-ranked ligands expressed in ECs and their inferred targets expressed in PC/vSMCs. Ligands and receptors are ordered by hierarchical clustering. **g** Scatter plots of AUC vs –log10(FDR) showing downregulated genes associated with selected GO terms in cKO PC/vSMCs. Red dots represent significantly downregulated genes (FDR<0.05); dashed line shows FDR = 0.05. Gene labels with red borders represent potential Runx1 target genes.

We next explored the impact of *Runx1* deletion in the hematopoietic niche and its possible effect on PC/vSMCs by performing scRNA-seq of *NG2-Cre;Runx1*^*fl/fl*^ cKO E11 AGMs (Fig. 4a, b). Cell populations were defined in a similar way to the WT AGM by using graph-based clustering and known marker distribution. This comparison revealed changes in the proportions of the different cell types between WT and cKO AGM, including a significant reduction in the proportion of cells associated with clusters 2 (Ery/EryP), 3 (IAHC), 6 (SNS), and 7 (SkMP) (Fig. 4c).

Changes in gene expression between WT and cKO *Cspg4*^*+*^*Runx1*^*+*^ cells were first investigated. We found that genes significantly downregulated in cKO *Cspg4*^*+*^*Runx1*^*+*^ cells were mainly associated with biological processes including translation, oxidative phosphorylation, cellular response to stress and mitochondria-related function (Fig. S4a–c). As deletion of Runx1 may have also affected *Runx1*^*−*^ PC/vSMCs, the transcriptome of all cKO *Cspg4*^*+*^*Acta2*^*+*^ PC/vSMCs with their WT counterpart was compared. Genes significantly downregulated in cKO *Cspg4*^*+*^*Acta2*^*+*^ PC/vSMCs had significant enrichment of biological processes including translation, smooth muscle cell differentiation, cytoskeleton, vasculogenesis and cell communication (Fig. 4d). One pathway essential to vasculogenesis is PDGF-B/PDGFRβ; we therefore applied NicheNet on our WT scRNA-seq data to predict ligand-receptor interaction between ECs and PC/vSMCs, focusing on PDGF-B-related genes. The highest scoring predicted interaction was between *Pdgfb*, a growth factor released by ECs, and *Nrp1* (Fig. 4e), a receptor known to control the differentiation/recruitment of mesenchymal stem cells and the stimulation of smooth muscle cell migration^37,38^.

The interaction between *Pdgfb* and *Pdgfrb* was also amongst the highest scoring interactions in both WT (Fig. 4e) and cKO (Fig. 4f). Additional interactions involve *Edn, Tgfb* or *Bmp* pathways, previously associated with a role in AGM hematopoiesis^39,40^. Interestingly, in cKO ECs, *Pdgfa*, another gene from the PDGF family, was no longer in the top 10 ranking ligands (Fig. 4f) possibly due to the downregulation of *Pdgfra* in cKO PC/vSMCs (Fig. 4f). Other genes including *Des*, *Angpt1*, *Gsk3b, Tcf21*, *Col1a1, Pcna, Ccnd3* and *Mcm7*, potential *Runx1* downstream target genes^41^, were also significantly downregulated (Fig. 4g, red boxes). The reduction in *Col1a1* expression suggests changes in the gene profile of the extracellular matrix (ECM). Indeed, additional ECM related genes were significantly downregulated in the cKO PC/vSMCs, such as *Sparcl1, Col3a1 and Col5a1* (Fig. 4g). Collectively, these data show that the genetic programme of PC/vSMCs in cKO AGM is modified upon Runx1 deletion and this involves changes in molecules that constitute the ECM of the aortic wall.

### Deletion of *Runx1* in NG2^+^cells leads to changes in the endothelial cell gene profile

**Fig. 5:**
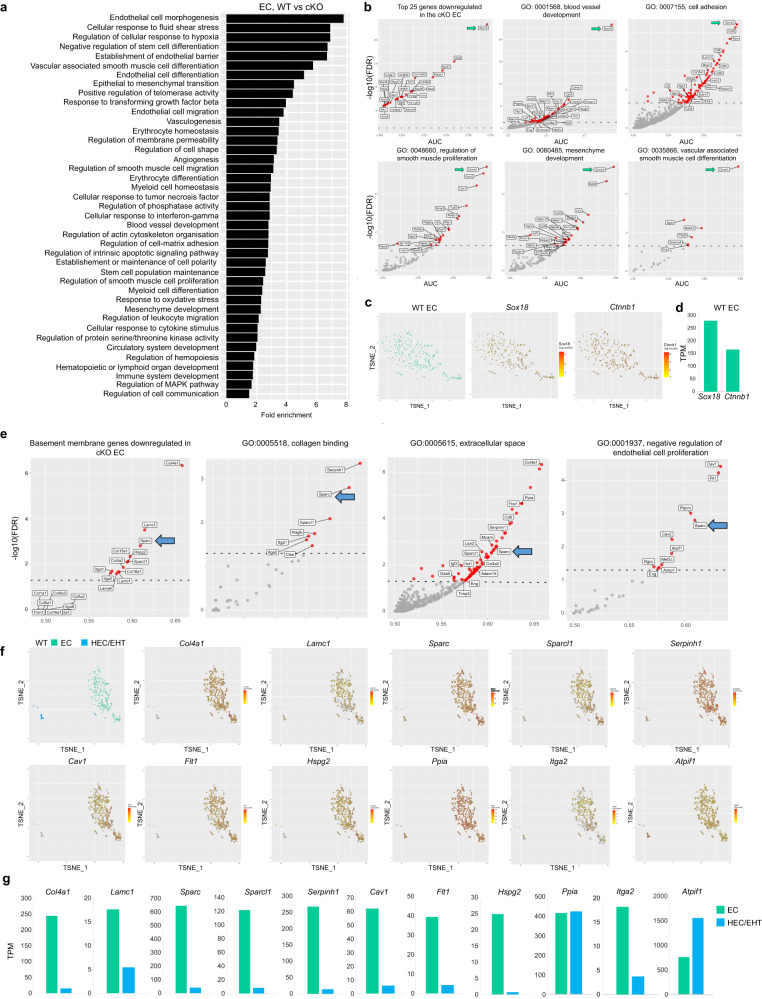
Deletion of Runx1 in NG2^+^cells results in changes in the extracellular matrix gene profile. **a** Barplot of fold enrichment for selected GO biological processes significantly overrepresented in genes significantly downregulated in cKO ECs compared to their WT counterparts. **b** Scatter plots of AUC vs –log10(FDR) showing downregulated genes associated with selected GO terms including blood vessel development and mesenchymal cells and vSMCs in cKO ECs. Red dots represent significantly downregulated genes (FDR < 0.05); dashed line shows FDR = 0.05. *Sox18* and *Ctnnb1* expression in WT ECs in both scRNA-seq (**c**, EC zoom and t**-**SNE plots) and bulk RNA-seq post-sort (**d**, TPM). **e** Scatter plots of AUC vs –log10(FDR) showing downregulated genes associated with selected GO terms including the basement membrane and extracellular matrix in cKO ECs. Red dots represent significantly downregulated genes (FDR < 0.05); dashed line shows FDR = 0.05. Selected genes that were altered in cKO ECs in **e** are shown in WT ECs in both scRNA-seq (**f**, EC and HEC/EHT zoom and t-SNE plots) and bulk RNA-seq post-sort (**g**, TPM). TPM: transcript per Million mapped reads values.

Endothelial cells share the same basement membrane with PC/vSMCs^42^. This, coupled with the transcriptomic changes in the cKO PC/vSMCs described above, suggest that the genetic programme of the adjacent ECs may have also been altered by *Runx1* deficiency in NG2^+^ cells. Although the number of endothelial cells in the *NG2-Cre:Runx1*^*fl/fl*^ cKO did not significantly change (Fig. 4a) and the formation of the dorsal aorta appeared to be unaffected (Fig. 1e), we investigated transcriptomic changes in ECs that could affect their function in vivo. As before, we performed differential expression analysis, followed by overrepresentation analysis on genes significantly downregulated in cKO ECs (Fig. 5a). Multiple GO biological processes were significantly overrepresented in these genes, with many related to EC development and angiogenesis; proliferation, migration and differentiation; response to hypoxia and fluid shear stress; as well as smooth muscle cell or mesenchymal cell development and hematopoiesis (Fig. 5a). Interestingly, we found that *Sox18* was the most downregulated gene in cKO ECs (Fig. 5b). *Col4a1*, the most abundant extracellular matrix associated gene, known to co-localise with *Sox18* in ECs in the mouse embryo^43^, was also found within the top 25 downregulated genes (Fig. 5b). *Sox18* and *Col4a1* were the most downregulated genes associated with the blood vessel development GO term, while other gene expression including *Cdh5*, *Pecam1*, *Sox17, Pdgfb, MCam* and *Notch* were also affected.

Genes associated with cell adhesion, regulation of smooth muscle cell proliferation and differentiation, along with mesenchyme development such as *Sox18* and *Ctnnb1* were also significantly downregulated in cKO EC (Fig. 5b, arrow). We confirmed that both *Sox18* and *Ctnnb1* are expressed by ECs in our single cell datasets (Fig. 5c) and next validated their expression in NG2^-^PDGFRβ^-^ckit^-^CD45^-^CD31^+^ Runx1^-^ purified ECs from E11 *Runx1-IRES-GFP* AGMs (Figs. 5d, S5a).

Some significantly downregulated genes associated with blood vessel development such as *Loxl2, Hspg2, Col4a2, Col15a1* and *Col18a1* (Fig. 5b) are also known to be associated to the ECM. Further analysis of endothelial extracellular matrix encoding genes previously described^44^ revealed that most of these genes were also significantly downregulated in cKO ECs (Fig. 5e). The expression of these genes in WT ECs at single-cell level (Fig. 5f) was confirmed post-sort at population-level (Fig. 5g) with most genes being highly expressed in ECs only. One of them was *Sparc* (Fig. 5e blue arrow, Fig. 5g), a central ECM secreted Ca^2+^-binding glycoprotein that interacts with many other ECM proteins including Col1 and Col4^45,46^. Among the SPARC family, *Sparcl1* (*Sparc-like 1*), known to bind to Col1^47^, was also found to be significantly downregulated in cKO ECs (Fig. 5b, c). Together, these analyses show that *Runx1* deficiency in NG2^+^cells leads to significant transcriptomic changes in endothelial cells including extracellular matrix related genes. We did not detect transcriptional changes in the *NG2-Cre;Runx1*^*fl/fl*^ cKO HEC/EHT cell cluster, although this observation is inconclusive due to the low number of cells captured.

### AGM scRNA-seq mostly captured *Runx1*^-^ IAHCs

Transcriptomic changes in vascular and perivascular cells may have also affected IAHCs. As hematopoietic cells are highly heterogeneous and progenitors were significantly affected (Fig. 1), we first explored WT IAHCs in more detail. Previous studies showed that IAHCs are composed of both *Runx1*^+^ and *Runx1*^-^ cells^28,48,49^ and we were able to confirm this by flow cytometry in *Runx1-IRES-GFP* AGMs (Fig. S5a). We also confirmed the expression of Runx1 in HEC/EHT and its absence in ECs by flow cytometry in *Runx1-IRES-GFP* E11 AGMs (Fig. S5a), in line with published work^50^. To validate their cell identity, we next purified and sequenced 243 Runx1 (GFP)^+^ and 27 Runx1 (GFP)^-^ IAHCs (NG2^-^PDGFRβ^-^ CD31^+^ckit^+^) as well as 5822 EC (Runx1^-^) and 248 HEC/EHT (Runx1^+^) cells from NG2^-^PDGFRβ^-^ ckit^-^ CD45^-^ CD31^+^ E11 *Runx1-IRES-GFP* AGMs (Fig. S5a) and performed bulk RNA sequencing (RNA-seq). The purity of the sort was first confirmed (Fig. S5b). While CD45 antibody was not used to isolate IAHCs (Fig. S5a), our bulk RNA-seq data (Fig. S5b) show that not all IAHC cells express *Ptprc* (CD45) in line with previous studies^48,49,51^, and seems to be present only when *Runx1* is expressed. Next, the identity of all sorted cell populations based on the expression of genes known to be expressed in these cells^15,35,48^ was confirmed (Fig. S5c). Interestingly, the transcriptomic profile of Runx1 (GFP)^+^ and Runx1(GFP)^-^ sorted IAHCs appears to be distinct. While *CD34*, *Gata2*, *Lmo2*, *Etv6*, and *Eglf7* are expressed in both Runx1(GFP)^+^ and Runx1(GFP)^-^ IAHCs at various levels, *Adgrg1*, *Gfi1*, *Myb* and *CD44* are mainly found in Runx1(GFP)^+^ IAHCs (Fig. S5c). Instead, as they also express *Tek, Kdr, Eng, Esam and Gata2* (Fig. S5c, d), Runx1(GFP)^-^ IAHCs are at the transcription level, closer to type-1 pre-HSCs^52,53^ or to recently described CD31^+^ckit^high^Gata2^medium^ IAHCs that are *Runx1*^-^*Ptprc*^-^^48^ with possible (micro)-niche role^54^. Our analyses confirm the heterogeneity of Runx1(GFP)^+/-^CD31^+^C-KIT^+^IAHCs in the E11 AGM at both protein and transcriptomic levels, and indicate that most IAHC cells captured in our full/unsorted AGM scRNA-seq are *Runx1*^-^.

### The genetic programme of cKO IAHCs is different from their WT counterpart

**Fig. 6:**
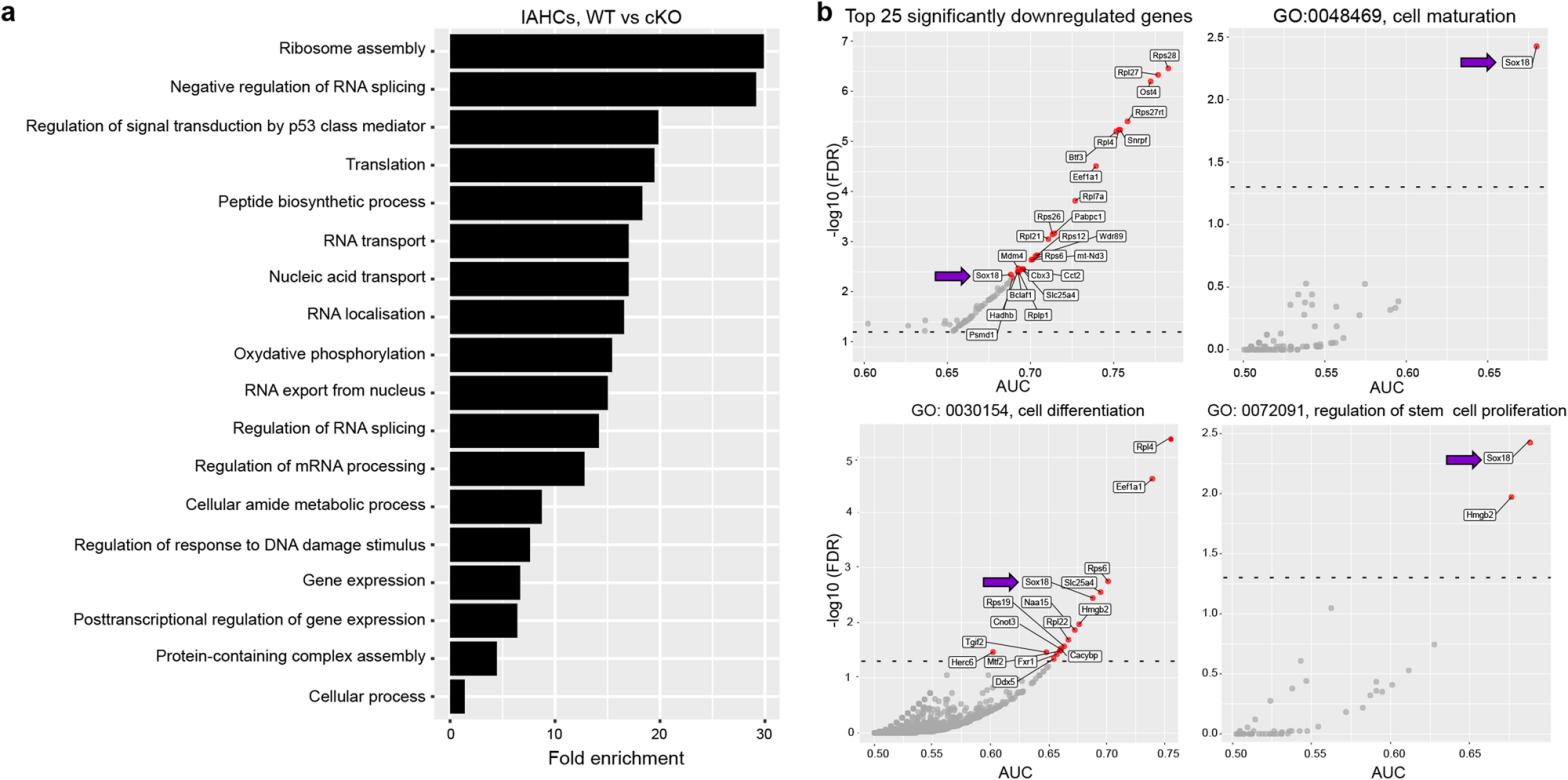
The genetic programme of cKO IAHCs is different from that of WT IAHCs. **a** Barplot of fold enrichment for selected GO biological processes significantly overrepresented in genes significantly downregulated in cKO HSPCs compared to WT HSPCs. **b** Scatter plot of AUC (representing strength of downregulation) vs –log10(FDR), showing the top 25 significantly downregulated genes (red circles) in cKO HSPCs. Scatter plots of AUC vs –log10(FDR) highlighting downregulated genes associated with Gene Ontology (GO) biological processes. Red dots found above the dashed line (corresponding to FDR = 0.05) represent significantly downregulated genes (FDR < 0.05).

To explore transcriptomic changes between WT and cKO IAHCs, differential expression analysis followed by overrepresentation analysis on genes significantly downregulated in cKO IAHCs was carried out (Fig. 6a). Several GO biological processes were significantly overrepresented in these downregulated genes, including ribosome assembly processes, regulation of translation, RNA transport and localisation, and others such as response to DNA damage, gene expression and cellular processes (Fig. 6a). In line with this, we found that the top 25 significantly downregulated genes in cKO IAHCs were mostly ribosomal protein coding genes from both *Rps* and *Rpl* families. Other genes in the top 25 are known to be required for transcriptional or translational initiation such as *Btf3, Pabpc1* and *Bclaf1* (Fig. 6b). Interestingly, one of the top significantly downregulated genes in the cKO was *Sox18* (Fig. 6b, arrow), previously reported to be expressed in both IAHCs and ECs in the mouse AGM^55^ and confirmed here by our WT scRNA-seq data (Fig. 2d). Furthermore, Sox18 has been transiently detected during early hematopoiesis in a model of embryonic stem cell differentiation in vitro, controlling early HP proliferation and maturation^56^. In line with this, further GO analysis revealed that *Sox18* is associated with cellular processes including cell maturation, cell differentiation and regulation of stem cell proliferation (Fig. 6b). The latter two GO terms are also associated with other genes significantly downregulated in cKO IAHCs such as *Hmgb2*, encoding a chromatin-associated non-histone protein involved in transcription and chromatin remodelling (Fig. 6b). This transcriptomic analysis shows that the deletion of *Runx1* in NG2^+^ PC/vSMCs within the AGM niche significantly alters the genetic programme of IAHCs.

### The reconstitution potential of bone marrow cKO HSCs is significantly reduced

**Fig. 7:**
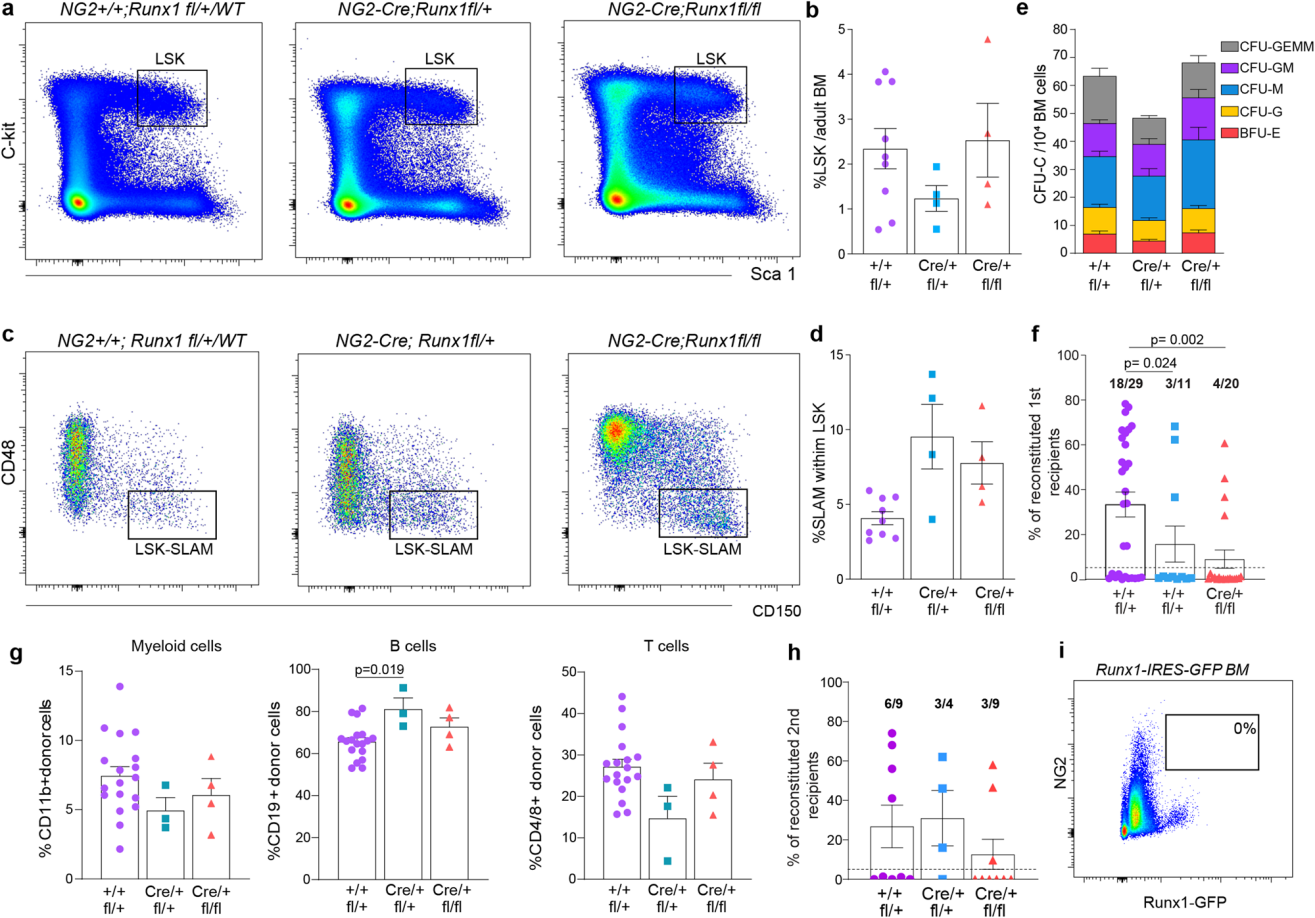
The reconstitution potential of bone marrow cKO HSCs is significantly reduced. **a**, **b** Representative plots and percentages of Lin^-^Sca1^+^cKit^+^ (LSK) and **c**, **d** LSK CD150^+^CD48^-^(SLAM) bone marrow (BM) cells by flow cytometry of WT/ *NG2+/+;Runx1*^*fl/+*^*,NG2+/+;Runx1*^*fl/fl*^ (*n* = 9), HET *NG2-Cre;Runx1*^*fl/+*^ (*n* = 4) and cKO *NG2-Cre;Runx1*^*fl/fl*^ (*n* = 4) adult mice is shown. **e** Colony-forming unit-culture (CFU-C) numbers per 10^4^ adult BM cells; *n* = WT/HET/cKO: 13/7/8 mice. *N* = 7 independent experiments. Data are mean ± SEM (Table S8). **f** Hematopoietic stem cell repopulating potential and donor chimerism of WT and mutant BM cells in vivo. 5 × 10^5^ BM donor WT, HET and cKO cells were injected into 29, 11 and 20 Ly5.1 HET recipients, respectively, with 18, 3 and 4 found to be reconstituted respectively (Table S3, *p* = 0.024 (WT/HET) and *p* = 0.002 (WT/cKO) by Z score test for 2 population proportions). Mice are reconstituted when ≥5% donor cells are found in the host peripheral blood; *p* = 0.002 (WT/cKO) by Kruskal-Wallis and Dunn’s post-hoc test (Table S4). **g** Histograms showing the contribution of CD45.2^+^CD45.1^-^ donor cells to myeloid cells (CD11b^+^Gr1^+/-^), B cells (CD19^+^) and T cells (CD4/8^+^) in all reconstituted host mice from (**f**). (*n* = WT/HET/cKO = 18/3/4), *p* = 0.019 (WT/HET) for B cells by one-way ANOVA and Tukey’s *post-hoc* test. **h** BM cells from selected reconstituted primary recipients (found in **f**) were transplanted into multiple irradiated secondary recipients. Mice are reconstituted when ≥5% donor cells are found in the host peripheral blood (Table S3–4). **i** Representative flow cytometric analysis plot of NG2 in *Runx1-IRES-GFP* adult BM (*n* = 6). All data are presented as Mean values+/-SEM. *N* = number of independent experiments; n = number of biological samples. Source data for **b**, **d**, **e**, **f**, **g** and **h** are provided as a Source Data file.

Despite the decrease in HPs and HSCs in cKO AGM, *NG2-Cre;Runx1*^*fl/fl*^ mice are born with no obvious defects and develop into adulthood. Because of this, we sought to explore the effect of *Runx1* deletion in NG2^+^ PC/vSMC on adult HSPCs. The presence of these progenitors in the adult bone marrow (BM) of mutant mice was analyzed by flow cytometry and compared to WT mice. No significant differences were found in either Lin^-^Sca1^+^cKit^+^ (LSK) (Fig. 7a, b) nor LSK CD150^+^CD48^-^(SLAM) cell frequencies (Fig. 7c, d) between cKO mice and WT controls. We performed HP assays and found that the frequencies of hematopoietic cell colonies were similar in all mutants and WT littermates (Fig. 7e, Table S8). To assess the capacity of these cells to reconstitute hematopoiesis in vivo, 5 × 10^5^ bone marrow cells harvested from all genotypes were transplanted into sub-lethally irradiated WT mice recipients. Compared to the control group in which 62.1% (18 out of 29) mice were reconstituted, mice injected with *NG2-Cre;Runx1*^*fl/+*^ or *NG2-Cre;Runx1*^*fl/fl*^ BM cells showed a significant reduction in the long-term reconstitution potential, with only 27.3% (3 out of 11, *p* = 0.024) and 20% (4 out of 20, *p* = 0.002) of transplanted mice being reconstituted respectively (Fig. 7f, Table S3). In addition, the percentage of donor chimerism was significantly reduced in the cKO group. On average, the donor chimerism with WT cells was 33% compared to the 16% and 9% observed when BM cells from NG2*-Cre;Runx1*^*fl/+*^heterozygous and *NG2-Cre;Runx1*^*fl/fl*^ cKO (*p* = 0.002) were injected respectively (Fig. 7f, Table S4). The remaining HSCs in the mutant *NG2-Cre;Runx1*^*fl/+*^ and *NG2-Cre;Runx1*^*fl/fl*^ adult BM are multilineage, showing similar contributions of donor cells to myeloid or lymphoid cell compartments (Fig. 7g), and self-renew (Fig. 7h). Interestingly, no NG2^+^Runx1(GFP)^+^ cells were detected in adult *Runx1-IRES-GFP* BM hematopoietic niches (Fig. 7i), suggesting that they are exclusive to the embryo and that the BM hematopoietic defect found in adults is developmentally driven.

## Discussion

In this study, we examined whether PC/vSMCs control AGM hematopoietic development in the mouse embryo in vivo. Previous work showed that pericytes support HSC maintenance in both the adult bone marrow and the fetal liver. We identified a subset of sub-aortic mesenchymal cells that co-express NG2 and Runx1 and display a sclerotome-derived vSMC transcriptomic profile. Analysis of scRNA-seq data and immunostaining observations showed that a subset of these cells also express CD146, a marker of PC/vSMC precursors in the AGM, recently described^21^. Adult CD146^+^ PCs were shown to support human and murine hematopoiesis^57,58^.

More recently, Runx1b:RFP^+^ AGM mesenchymal cells were shown to support hematopoiesis in vitro^35^. In this study, authors performed aggregation cultures of hematopoietic cells with sorted stromal cells from E10.5 AGM in the presence or the absence of Runx1b:RFP^+^ cells, and found that the CFU potential was decreased in the absence of Runx1b:RFP^+^ cells. While this shows that at this stage Runx1b:RFP^+^ mesenchymal cells support hematopoiesis in vitro^35^, the direct identification of the precise mesenchymal cell population expressing Runx1 with a functional role in the mouse hematopoietic AGM niche in vivo had not yet been elucidated. Similarly, scRNA-seq showed that the Runx1b:RFP^+^ mesenchymal cell population is sub-divided into high *Pdgfra*^+^ and *Acta2*^+^ cell subsets^35^. Whether only one of these individual populations or both support mouse HSPC development in vivo or in vitro remains unclear.

We hypothesised that NG2^+^Runx1^+^ PC/vSMCs control in vivo HSPC development in the mouse embryo. Indeed, conditional deletion of Runx1 in NG2^+^ cells significantly reduced the number of HSPCs in the AGM together with the number of reconstituted mice by donor cKO AGM cells in vivo. Importantly, neither antibody-selected NG2^+^Runx1(GFP)^+^ cells nor lineage-traced NG2-derived daughter cells in the AGM gave rise to CFUs, demonstrating a functional role for Runx1 as a positive hematopoietic niche regulator in the PC/vSMCs perivascular layer (Fig. S6).

We found that the number of mice that reconstituted hematopoiesis upon transplantation with *NG2-Cre;Runx1*^*fl/fl*^ cKO BM cells and the level of donor chimerism was significantly decreased. Interestingly, the cKO mice are born with no apparent hematopoietic defect. This is not a surprise. Yokomizo et al. recently showed that the embryonic hematopoietic system can be maintained with minimal involvement of HSCs^59^. Furthermore, Dignum et al., demonstrated that in vitro, multipotent progenitors and HSCs arise independently from hemogenic endothelial cells in the mouse embryo^60^. In line with these studies, we showed that the HP numbers in the placenta, FL and adult BM are not affected in *NG2-Cre;Runx1*^*fl/fl*^ cKO mice. This suggests that an HSC-independent production of these progenitors is sufficient for the mice to survive in addition to the remaining cKO BM HSCs that are multilineage and self-renew. Furthermore, this also suggests that the development of HSC-independent progenitors does not require NG2^+^Runx1^+^ niche cells. However, we cannot rule out that the presence of HSCs in the cKO BM may be due to the low Cre recombination efficiency of the *NG2-Cre* mice used, as in average, only 20% of NG2^+^ cells expressed tdTomato in the AGM.

We next discovered that changes in the in vivo PC/vSMC genetic programme, modified both IAHC and EC transcriptomic profiles in the E11 AGM. Although we did not capture all IAHCs by scRNA-seq, we compared the transcriptional profile of mainly Runx1^-^ IAHCs between cKO E11 AGMs and its WT littermate controls. We found that one of the top 25 significantly downregulated genes in the cKO IAHCs was *Sox18*. Ectopic expression of SOX18 was previously linked to the self-renewal potential of HPs in the embryonic system in vitro^56^, suggesting a role for NG2^+^Runx1^+^ niche cells in HSPC maintenance.

In addition to SOX18, two other SOX transcription family members, SOX7 and SOX17, were reported to be involved in both blood vessel development and hematopoiesis^61^. We investigated their expression in our datasets and found that all three transcription factors were highly expressed in IAHCs and ECs as previously shown^43,55,56^. In ECs, *Sox17* is known to play a role in the maintenance of clusters containing HSCs^55^. We discovered that in the *NG2-Cre;Runx1*^*fl/fl*^ cKO AGM, both *Sox18* and *Sox17* were significantly downregulated in ECs, specifically driving the enrichment of blood vessel development-related processes. Indeed, we observed a significant reduction of IAHC in the *NG2-Cre;Runx1*^*fl/fl*^ cKO AGM alluding to the importance of the fine regulation that PC/vSMCs and ECs play in controlling one another as well as HSPCs. Together with changes in PC/vSMCs affecting ECs, an opposite effect was also observed. We found that genes associated with the regulation of smooth muscle cell proliferation and differentiation, or mesenchymal development were significantly downregulated in cKO ECs. One of these genes, *Pdgfb*, detected in a significantly smaller proportion of cKO ECs (42% in cKO compared to 59% in the WT, *p* = 0.0001) but also with significantly lower expression (FDR = 0.02), was shown to mediate the vSMC phenotype switch from a contractile to a synthetic/proliferative state, by regulating *Sox9*^62^ or *Yap1*^63^ gene expression. In our cKO scRNA-seq analysis, *Yap1* was downregulated in both ECs and PC/vSMCs, although the difference in PC/vSMCs was not significant. However, major proliferation-associated genes were significantly downregulated in the cKO PC/vSMCs, suggesting a premature switch of these cells towards a more mature phenotype^21^. Whether PC/vSMC proliferation was exclusively due to *Pdgfb* decrease is not clear. Some of these proliferation-associated genes significantly downregulated in PC/vSMCs, such as *Pcna, Ccnd7* and *Mcm7*, are known downstream target genes of *Runx1* and thus, changes in the proliferation status of PC/vSMCs may be intrinsic. The upregulation of genes that characterise a more mature/contractile smooth muscle cell phenotype such as *Cnn1* or *Tagln*, in cKO PC/vSMCs, was not significant. This could be due to the presence of CD146 in these cells, known to prevent vSMC differentiation^21^, and expression of which remained unchanged in the cKO PC/vSMCs.

Our scRNA-seq analysis also revealed that *Sparc* is significantly downregulated in cKO ECs in the E11 AGM and we found that many collagen genes, known to interact with *Sparc*, were downregulated in ECs and/or PC/vSMC. Reduction in collagen concentration and compromised collagen fibril formation has been found in SPARC-KO mice, suggesting a critical role for SPARC in the ECM assembly^64^. In addition, the depletion of SPARC in flies results in the absence of Col4 in the basal lamina during development, leading to embryonic death^65^. Interestingly, our ongoing preliminary data suggests that SPARC plays a role in HSC maintenance or maturation ex vivo. When added in explant culture, SPARC is able to rescue the *NG2Cre;Runx1*^*fl/fl*^ cKO AGM hematopoietic defect upon transplantation into irradiated recipient mice (data not shown).

In conclusion, we have described and characterised the regulatory potential of NG2^+^Runx1^+^ perivascular cells in the HSC generating niche in vivo. Our study shows the crucial role of pericytes/vSMCs in maintaining the AGM niche, and thus the regulation of HSC generation. These findings offer a deeper molecular understanding of the complex regulation of the AGM niche providing novel and important observations that may lead to ways to resolve the complexity of HSC generation.

## Methods

### Animals and embryo generation

All experiments were performed under a Project Licence granted by the Home Office (UK), approved by the University of Edinburgh Ethical Review Committee and conducted in accordance with local guidelines. Mice were bred and housed at the Centre for Regenerative Medicine, Edinburgh, UK under a 12-h light/12-h dark cycle mimicking circadian rhythm and fed a chow diet and water *ad libitum. NG2Cre* transgenic mice (Jackson Laboratory, B6.Cg-Tg(Cspg4-cre/Esr1*)BAkik/J) were backcrossed into the C57BL/6J background and maintained as heterozygous. *Runx1*^*fl/fl*^ mice (from Professor Nancy Speck, available in Jackson Laboratory), *R26-TdTomato mice* (Jackson Laboratory, Gt(ROSA)26Sor^tm14(CAG-tdTomato)Hze^) and *Runx1-IRES-GFP* mice (from James Downing, Runx1^tm4Dow^) are on a C57BL/6J background and were maintained as homozygous. C57BL/6J (Ly5.2) WT mice (CD45.2+CD45.1-) and Ly5.1 homozygous (CD45.2-CD45.1+) and heterozygous (CD45.2+CD45.1+) inbred mice were provided by our animal facilities. All mice used in this study were males and females mixed between 3 and 6 months of age. The day of vaginal plug detection was designated as developmental day (E)0.5.

### Genotyping

DNA was extracted from the remaining embryonic organs that were not used in experiments. Tissue was placed in a solution containing 100 µl of Extraction solution (Sigma, E7526) and 25 µl of Tissue preparation solution (Sigma, T3073). Samples were incubated in a shaking block at 55 °C for 10 min, followed by DNA denaturation for 3 min at 95 °C. After denaturation, 100 µl of Neutralisation solution B (Sigma, N3910) was added to each sample. Primers per mouse line were used as follow: **NG2Cre**: mNG2cre forward: GCG GTC TGG CAG TAA AAA CTA TC, mNG2Cre reverse: GTG AA ACA GCA TTG CTG TCA CTT; **Runx1fl:** mEx4_intF563_F: CCC ACT GTG TGC ATT CCA GAT TGG, mEx4_R837_R: GAC GGT GAT GGT CAG AGT GAA GC and CAC CAT AGC TTC TGG GTG CAG, **Runx1-IRES-GFP**: AML1-GFP USA F: GTC CAG GAG CGC ACC ATC TTC TTC, AML1-GFP USA R: GTA CAG CTC GTC CAT GCC GAG AGT; **R26-TdTomato:** ROSA WT_F: AAG GGA GCT GCA GTG GAG TA, ROSA WT_R: CCG AAA ATC TGT GGG AAG TC, tdTomato_F: CTG TTC CTG TAC GGC ATG G, WPRE_R: GGC ATT AAA GCA GCG TAT CC.

### In vitro Colony Forming Unit-Culture assays (CFU-C)

Except for the fetal liver (FL) which was only mechanically disrupted into single-cell suspension, other embryonic organs were dissected and dissociated with collagenase type I (Sigma, C0130, 0.12% v/v) in PBS for 45 min (AGM, head) and 75 min (placenta, yolk-sac/YS) at 37 °C, mechanically dissociated into single-cell suspensions and washed with 10% FCS in PBS. Adult bone marrow (BM) cells were flushed from wild type, heterozygous and knock-out femur/tibia bones and lysed with ammonium chloride solution (Stem Cell Technologies 07850) for 12 min at room temperature (RT). AGM (1 embryo equivalent (ee)/dish for E10 and E10.5 and 0.33ee/dish for E11), FL (0.5ee/dish for E10.5 and 0.05ee/dish for E11), head (0.5ee/dish for E10 and 0.33ee/dish for E11), fetal placenta, YS (0.5ee/dish for E10 and 0.33ee/dish for E11) and 10^4^ BM cells/dish were plated in methylcellulose (MethoCult GF M3434, Stem Cell Technologies Inc.) supplemented with 1% PS in 35 mm Petri dishes (Falcon 1008). Dishes were then incubated for 10–12 days at 37 °C, 5% CO_2_ in a humidified glass chamber and the different CFU-C types were identified based on their morphology and then counted. CFU-C types are defined as: blast forming unit erythroid progenitors (BFU-E), colony forming unit-granulocyte progenitors (CFU-G), macrophage progenitors (CFU-M), granulocyte and macrophage progenitors (CFU-GM), and the most immature granulocyte, erythrocyte, monocyte and megakaryocyte progenitors (CFU-GEMM).

### In vivo transplantation assays

Female, Ly5.1 heterozygous (CD45.1^+^CD45.2^+^) recipient mice were sub-lethally irradiated with a split dose of 9–9.6 Gy, 3 h apart and single-cell suspensions of E11 AGM or E11 FL obtained as described above, were injected intravenously via the tail vein (1ee/mouse) together with 2 × 10^4^ bone marrow (BM) helper cells from Ly5.1 homozygous (CD45.1^+^CD45.2^-^) mice. To assess donor cell chimerism, peripheral blood was taken at 1- and 4-months post-transplantation and analysed for the presence of CD45.1^-^CD45.2^+^ cells and multilineage reconstitution by flow cytometry. Red cells were first lysed with ammonium chloride solution for 12 min at RT and incubated for 30 min at 4 ^°^C in the dark with the following antibodies from Biolegend: CD4 PE (1:5000, 130310), CD8a PE (1:500, 100708), CD11b/Mac-1 APC (1:1000, 101212), CD19 APC-Cy7 (1:1000, 115530), CD45.1 FITC (1:1000, 110706), CD45.2 Pacific Blue (1:1000, 109802) and Gr-1/Ly-6G/C PE-Cy7 (1:2000, 108416). Cells were then washed, re-suspended in 300 µl of 2% FCS in PBS and analysed by flow cytometry. Sytox AAD (1:1000, Thermofisher) was used for dead cell exclusion. Mice with ≥5% CD45.1^-^CD45.2^+^ cell chimerism were considered positively repopulated. In some cases, following 4 months analysis, successfully reconstituted mice were sacrificed and the BM cells from both legs were harvested to perform secondary transplantations. Cells from one primary recipient were transplanted into two secondary irradiated recipients. Peripheral blood from secondary recipients was analysed after 1 and 4 months as described above. For primary transplantations with adult BM from all genotypes, 5 × 10^5^ cells were injected intravenously via the tail vein into sub-lethally irradiated mice without helper cells being co-transplanted and analysed as described above.

### Immunohistochemistry

Embryos and placentas were dissected, and a small piece of tissue was taken for genotyping. Embryos and placentas were fixed in 2% paraformaldehyde (PFA) for 20 min on ice, and washed three times with PBS, 10 min each. Tissues were next dehydrated overnight (O/N) in 20% sucrose/PBS at 4 °C. Embryos and placentas were then embedded in optimum cutting temperature (OCT) medium, frozen in 100% ethanol cold vapours in dry ice, and stored in −20 °C until used. Sections 10 µm thick were cut using a Bright OTF5000 cryostat. Slides were fixed with 100% cold methanol or 1:1 cold methanol: acetone for 5 min at RT, washed three times, 5 min each, with PBS and incubated with blocking solution (Spring Bioscience) for 30 min or 10% or 5% goat serum in PBS 1 h in a humidified chamber at RT. The following unconjugated primary antibodies were incubated overnight at 4 °C: NG2 (1:100, Rabbit polyclonal, Millipore, ab5320), NG2 (1:50, Rat anti-mouse, R&D systems, MAB6689), Rabbit anti-RFP to detect TdTomato (1:100, Rockland, 600-401-379), CD45 (1:100, Goat anti-mouse, R&D systems, AF114), F4/80 (1:50, Rat anti-mouse, Abcam, ab6640), and Runx1,2,3 (1:100, Rabbit anti-mouse, Abcam, ab92336). For sections incubated with Runx1, a previous step of incubation with 0.5% Triton (TritonX-100, Acros Organics) was necessary, followed by washes with PBS and 0.5% Triton. The conjugated primary antibodies were incubated for 2 h at RT: CD31 (1:50, Biotinylated rat anti-mouse, BD Pharmingen, 553371), CD146-AF488 (1:100, Rat anti-mouse, Biolegend, 134707), αSMA-Cy3 (C6198) or αSMA-FITC (F3777) (1:100, Mouse anti-mouse, Sigma). Slides were then washed three times, 5 min each in PBS and sections incubated with secondary antibodies (1:250, Invitrogen) for 2 h at RT: goat anti- rabbit Alexa 488 (A11008), Alexa 546 (A11035), Alexa 647 (A21244), goat anti-rat Alexa 594 (A11007), donkey anti-goat Alexa 594 (A11058), chicken anti-rat Alexa 647 (A21472), goat anti-rabbit Alexa 594 (Life Technologies, A11012) or with Streptavidin FITC (1:100, BD Pharmingen, 554060), Streptavidin 555 (1:300, Invitrogen, S32255) or Streptavidin 647 (1:250, Life Technologies, S21374) for 30 min at RT. Slides were washed three times for 5 min each in PBS and sections stained with DAPI (1:500; Molecular Probes) for 15 min at RT in a dark, humidified chamber. Slides were next washed three times 5 min each in PBS and mounted using Fluoromount-G (Southern Biotech). Images were taken using an inverted widefield fluorescence microscope (Zeiss Observer) with the Zen 2.3 pro (blue edition) software, and analysed either using Fiji/ImageJ 1.52e software or Zen Blue software and Huygens Professional software (v19.10) for stitching and deconvolution.

### Flow cytometry

Tissues were dissected from E9 to E11 mouse embryos, digested mechanically (fetal liver, FL) or in collagenase type I (AGM; yolk sac,YS; placenta; head) as described above. For flow cytometry analysis, cells were incubated for 30 min at 4 °C in the dark with NG2 Cy3 (1:100, Millipore, ab5320c3), NG2 AF488 (1:100, Millipore, ab5320a4) or CD45 PerCpCy5.5 (1:400, BD Pharmingen, 550994). Samples were then washed twice in PBS/FCS and analysed using a BD LSR Fortessa 4 laser (BD Biosciences). To perform CFU-C assays, NG2^+/-^ cells were sorted using a BD FACSAria Fusion with BD FACS Diva Software V8.0.l (BD Biosciences) and seeded for 10 to 12 days in methylcellulose as described above. For NG2-Cre; R26TdTomato, E11 AGMs were sorted based on TdTomato expression. As all E11 AGM HSPCs have been reported to express cKit^15^, E11 AGM cells were also stained with cKit (1:500, BD Horizon, 562609) for further enrichment. Sorted cells were seeded in Methocult or transplanted as described above. For LSK and LSK-SLAM analysis, the four bones from adult mouse limbs were dissected and the bone marrow flushed with 10%FBS/1%P/S/2 mM EDTA (Lonza). Red cells were lysed with ammonium chloride solution for 12 min at RT and the samples incubated for 30 min at 4 °C in the dark with the following antibodies: BD Biosciences biotin CD4 (1:600, 553648), CD5 (1:800, 553018), CD8a (1:800, 553028), CD11b/Mac-1 (1:200, 553309), CD45R/B220 (1:200, 553086), Gr-1/Ly-6G/C (1:100, 553125), Ter119 (1:50, 553672) and Biolegend: c-kit APC (1:200, 105812), Sca-1 APC-Cy7 (1:200, 122514), CD48 PE (1:800, 103406), CD150 PeCy7 (1:200, 115914). Samples were then washed twice in PBS/FCS and further incubated with Streptavidin PerCP (1:200, Biolegend, 405213) for 30 min at 4 °C in the dark. Cells were washed and analysed using BD LSR Fortessa 4 laser (BD Biosciences). Bone marrow from adult Runx1(GFP) mice stained with NG2-Cy3 antibody was analysed using either BD LSR Fortessa 4 laser (BD Bioscience) or Novocyte Flow Cytometer (ACEA Biosciences), and NovoExpressTM Software (1.5.0) and the softwares FlowJo X (v10.0.7).

### Wholemount immunostaining

Wholemount embryo immunostaining and three-dimensional imaging were performed as previously described^66^. Embryos were first separated from the placenta and the YS, fixed in 2% PFA/PBS for 20 min on ice and washed 3 times with PBS for 10 min each. The YS was taken for genotyping. Embryos were next dehydrated twice with 50% methanol in PBS for 10 min at 4 °C, twice with 75% methanol in PBS for 10 min at 4 °C and finally with 100% methanol for 10 min at 4 °C. Embryos were further dissected to remove the head, limb buds, and one side of the body wall, rehydrated in 75% methanol and 50% methanol for 10 min each at 4 °C and then incubated with biotin and avidin blocking solution (Life Technologies) for 15 min each at RT. Lastly, the embryos were incubated with BSA/PBS-MT 10% (w/v) BSA, 1% (w/v) of skim milk and 0.4% (v/v) of TritonX in PBS for 1 h at 4 °C. Embryos were stained for several days with unconjugated cKit (1:500, BD Bioscience, 553352), biotinylated CD31 (1:500, BD Pharmingen, 553371), unconjugated NG2 (1:500, R&D Systems, MAB6689), Runx1,2,3 (1:250, Abcam, ab92336) and αSMA FITC (1:500, Sigma, F3777). Secondary antibody Alexa647 (1:2500, Invitrogen, A21244) and Streptavidin Cy3 (1:5000, Sigma, S6402) were used to detect cKit and CD31, respectively. Secondary antibody Alexa594 (1:2500. Invitrogen, A11007) and Alexa647 (1:2500, Invitrogen, A21472) were used to detect NG2 and Runx1 respectively. Embryos were then transferred to glass containers where the methanol was replaced by 50% BABB/methanol (1 part benzyl alcohol with 2 parts benzyl benzoate in methanol) 4 times and then with 100% BABB until becoming completely clear. Embryos were eventually mounted on a small chamber and imaged using a Leica SP8 confocal microscope and Leica Application Suite X software (v3.5.5.19976).

### Counting of Intra-aortic hematopoietic clusters

Wholemount immunostaining and three-dimensional imaging of E10.5 WT and cKO mouse embryos stained for cKit and CD31 were used to count IAHCs. Groups of more than one cKit^+^CD31^+^ or CD31+ cell attached to the ventral wall of the dorsal aorta were considered as one cluster. The total number of clusters were counted and compared between WT and cKO embryos.

### Single-cell RNA sequencing and analysis

#### Cell preparation

E11 AGM cells were dissected and dissociated using collagenase type 1 as described above. Single-cell suspensions were washed, and the pellets were resuspended in PBS 10%FCS and 1% PS. *Library preparation and sequencing.* Cells were processed through the 10× Genomics Chromium Single Cell Platform using the Single Cell 3’ Library and Gel Bead Kit v3 (10X Genomics) as per the manufacturer’s instructions. In brief, cells were counted and assessed for viability using a Bio-Rad TC20 then loaded at a concentration for the recovery of 7000 cells and processed through the 10X Chromium Controller. Single-cell libraries were obtained according to manufacturer protocol. RNA concentration was obtained using Quibit RNA HS (Thermo-Fisher) and the quality of the libraries was verified using the LabChip GX24 (Perkin Elmer). Libraries were sequenced on an Illumina Novaseq 6000 S1 lane. The sequencing run met Illumina’s quality metrics. Data were demultiplexed using 10X Genomics Cell Ranger ‘mkfastq’ command. *Data analysis*. Alignment of scRNA-seq data and barcode counting was performed using Cell Ranger ‘count’ (v3.1.0) with reference dataset mm10/GRCm38-3.0.0. The unfiltered UMI count matrix from Cell Ranger was used as input for downstream analysis following the OSCA Bioconductor workflow^67^. EmptyDrops^68^ was used to distinguish cells from droplets predicted to contain only ambient RNA, calling 17,419 (WT) and 19,047 (cKO) cells at the default FDR of 0.1%. Quality metrics (library size, number of expressed features and percentage of mitochondrial reads) were computed for the called cells using the scater Bioconductor package (v1.14.6)^69^. Cells with any quality metric more extreme than 3 median absolute deviations from the median were filtered out; 15,220 (WT) and 17,280 (cKO) cells were retained for downstream analysis. Count matrices for both samples were log-normalised; size factors for all cells were computed by deconvolution^70^. Technical noise was modelled using a Poisson-based trend, serving as a lower bound for the variance of endogenous genes. Dimensionality reduction for denoising was performed with principal component analysis (PCA) using the modelled trend. Shared-nearest neighbour graphs were constructed using the PCA-reduced datasets and used as input to the Walktrap community finding algorithm to obtain cell clusters. These clusters were used as an initial approximation to known cell types; we refined this approximation based on the distribution and expression levels of literature-derived marker genes. For each sample, doublet scores were computed for each cell by performing an in silico simulation of doublet cells from cell expression profiles^71^; no doublet-driven clusters were detected. Data from both samples were integrated using a fast version of the mutual nearest neighbours method from the batchelor Bioconductor package (v1.2.4)^72^. Projection of all cells into a shared *t*-SNE space confirmed the consistency of the cell-type annotations. Differential expression was computed by Wilcoxon rank sum test between appropriate groups using the scran Bioconductor package (v.1.18.7)^70^. Genes with FDR < 0.05 were considered to be significantly differentially expressed. Enrichment analysis with the PANTHER classification system^73^ was used to identify GO biological processes overrepresented among significantly differentially expressed genes. Fold enrichment of GO biological processes was computed by Fisher’s Exact Test with FDR correction; processes with FDR < 0.05 were considered to be significantly enriched. We used AmiGO (v.2.5.13)^74^ to perform additional data mining of GO terms. Prediction of ligand-receptor interactions was computed using the nichenetr (v.1.0.0) R package. We considered genes to be expressed in either WT ECs or PC/vSMCs if they had detectable expression in at least 25% of cells of that cell type. The top 10 ligands (‘prioritised ligands’) expressed in ECs were chosen based on Pearson correlation coefficient; target genes expressed in PC/vSMCs were inferred using these ligands. Ligand-receptor network inference was used to predict and score potential interactions. Cell lineage inference was performed on WT *NG2*^*+*^*Runx1*^*+*^ cells using the Slingshot Bioconductor package (v.2.2.1). We defined a discrete cluster of NG2^+^Runx1^+^Acta2^-^ cells as the cluster of origin. For pseudotime plots, cells were ordered according to the principal curve defined by Slingshot and log normalised expression of each cell plotted. Trend lines and standard error bounds were computed by loess local polynomial regression fitting.

### Bulk RNA sequencing and analysis

E11 embryos were obtained from Runx1-GFP/+ mice mating and AGM cells stained with NG2 Cy3 (1:100, Millipore, ab5320c3), PDGFRβ APC (1:100, Biolegend, 136008), CD31 PECy7 (1:4000, eBioscience, 25-0311-82), CD45 PerCypCy5.5 (1:400, BD Pharmingen, 550994) and ckit BV421 (1:500, BD Horizon, 562609) for 30 min at 4 °C, washed, centrifuged, then resuspended in PBS/FCS/PS for analysis and cell sorting. Cells were purified using BD FACS ARIA Fusion cell sorter (BD Bioscience) and Sytox AAD was used to select viable cells. Cells were sorted and collected directly into 10–20 µl of lysis buffer containing Nuclease-free water (Ambion AM9930), 0.2% Triton and 1/20 RNAse inhibitor (Thermoscientific 10777019). Full-length cDNA was generated from 2.3 µl of this cell lysate using the Smarter2 procedure as described^75^. Sequencing libraries were generated from 500 pg of cDNA with Illumina’s Nextera XT sample prep kit (Illumina Inc., U.S.A) and according to the Illumina TruSeq Rapid v2 protocol on the HiSeq2500 with a single read 51 bp and dual 9 + 9 bp index (Illumina Inc., U.S.A). Reads were aligned against the GRCm38 reference using HiSat2 (version 2.0.4). We called gene expression values using GENCODE M19 gene annotation file and the union mode in the Bioconductor Genome Alignments Package (v1.8.1).

### Statistical tests

GraphPad Prism 7 was used to perform all statistical tests. Normal distribution was assessed using the Shapiro-Wilk test. Based on data distribution, either one-way ANOVA (parametric) or Kruskal-Wallis (non-parametric) with Tukey’s and Dunn’s post-hoc test, respectively, were used. When required, unpaired *t* test was used.

### Reporting summary

Further information on research design is available in the Nature Portfolio Reporting Summary linked to this article.

### Supplementary information

Supplementary Information

Reporting Summary

### Source data

Source Data

## Data Availability

The scRNA-seq data and the bulk RNA-seq data from this study are publicly available on the Gene Expression Omnibus (GEO) database under accession numbers GSE178981 and GSE229850 respectively [https://www.ncbi.nlm.nih.gov/geo/query/acc.cgi?acc=GSE229850]. Source data are provided in this paper for all graph bars in the manuscript (Figs. 1d/h–k, 7b/d–h, S1g, S2a–e, and S3b–e) and for Fig. 2e. Source data are provided with this paper.
