# Supplementary material for: Runx1+ vascular smooth muscle cells are essential for hematopoietic stem and progenitor cell development in vivo
Source: Nat Commun. 2024 Feb 23;15:1653. doi: 10.1038/s41467-024-44913-z (PMC10891074; doi:10.1038/s41467-024-44913-z)
Supplement: Supplementary file 1 — Supplementary Information [file 41467_2024_44913_MOESM1_ESM.pdf]

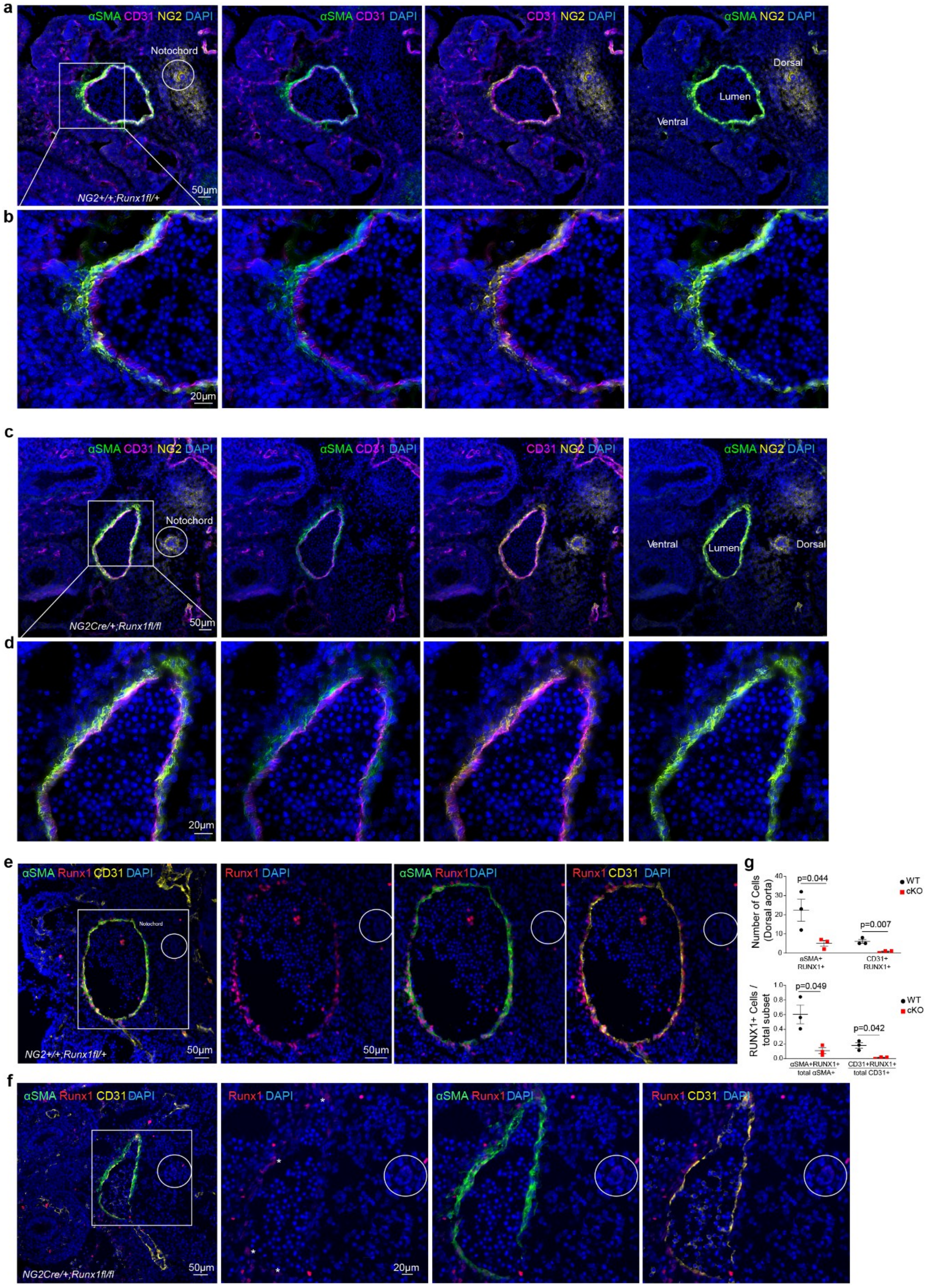
a
αSMA CD31 NG2 DAPI
Notochord
αSMA CD31 DAPI
CD31 NG2 DAPI
αSMA NG2 DAPI
Dorsal
Lumen
Ventral
50µm
NG2+/+;Runx1fl/+
b
20µm
c
αSMA CD31 NG2 DAPI
Notochord
αSMA CD31 DAPI
CD31 NG2 DAPI
αSMA NG2 DAPI
Ventral
Lumen
Dorsal
50µm
NG2Cre/+;Runx1fl/fl
d
20µm
e
αSMA Runx1 CD31 DAPI
Notochord
Runx1 DAPI
αSMA Runx1DAPI
Runx1 CD31 DAPI
50µm
NG2+/+;Runx1fl/+
50µm
g
Number of Cells (Dorsal aorta)
p=0.044
p=0.007
aSMA+ RUNX1+
CD31+ RUNX1+
WT
cKO
RUNX1+ Cells / total subset
p=0.049
p=0.042
αSMA+RUNX1+ / total αSMA+
CD31+RUNX1+ / total CD31+
WT
cKO
f
αSMA Runx1 CD31 DAPI
Runx1 DAPI
αSMA Runx1 DAPI
Runx1 CD31 DAPI
50µm
20µm
NG2Cre/+;Runx1fl/fl

Figure S1. Characterisation of *NG2$^{+/+}$;Runx1$^{fl/fl}$* WT and *NG2-Cre;Runx1$^{fl/fl}$* cKO E11 AGM by immunohistochemistry on frozen sections. Low magnification **(a, c)** and high magnification **(b, d)** images showing the expression of NG2 (yellow) and αSMA (green) in combination with CD31 (purple) in both WT *NG2+;Runx1$^{fl/+}$* **(a, b)** and cKO *NG2-Cre;Runx1$^{fl/fl}$* **(c, d)** dorsal aorta and around the notochord. N = 2 independent experiments with WT/cKO n=2/2. Single, double, and triple marker combinations of αSMA, Runx1 and CD31 shown in the WT **(e)** and cKO **(f)** AGM. The notochord is encircled. DAPI stains all nuclei (blue). N=2 independent experiments with WT/cKO n=2/2. **g.** Quantification of αSMA^{+}Runx1^{+} (p=0.044, Unpaired t-test, two-tailed) and CD31^{+}Runx1^{+} (p=0.007, Unpaired t-test, two-tailed) cells and respective ratios αSMA^{+}Runx1^{+}/total αSMA^{+} cells (p=0.049, Unpaired t-test with Welch's correction, two-tailed) and CD31^{+}Runx1^{+}/total CD31^{+} cells (p=0.042, Unpaired t-test with Welch's correction, two-tailed) in the dorsal aorta in both WT/ *NG2+;Runx1$^{fl/+}$* and cKO *NG2-Cre;Runx1$^{fl/fl}$* dorsal aorta transversal sections (n=2 samples/genotype, 1-2 technical replicates/sample, N=2 independent experiments). Both absolute numbers (up) and ratios (bottom) are shown. All data are presented as Mean values+/-SEM. N = number of independent experiments; n = number of biological samples. Source data for j are provided as a Source Data file.

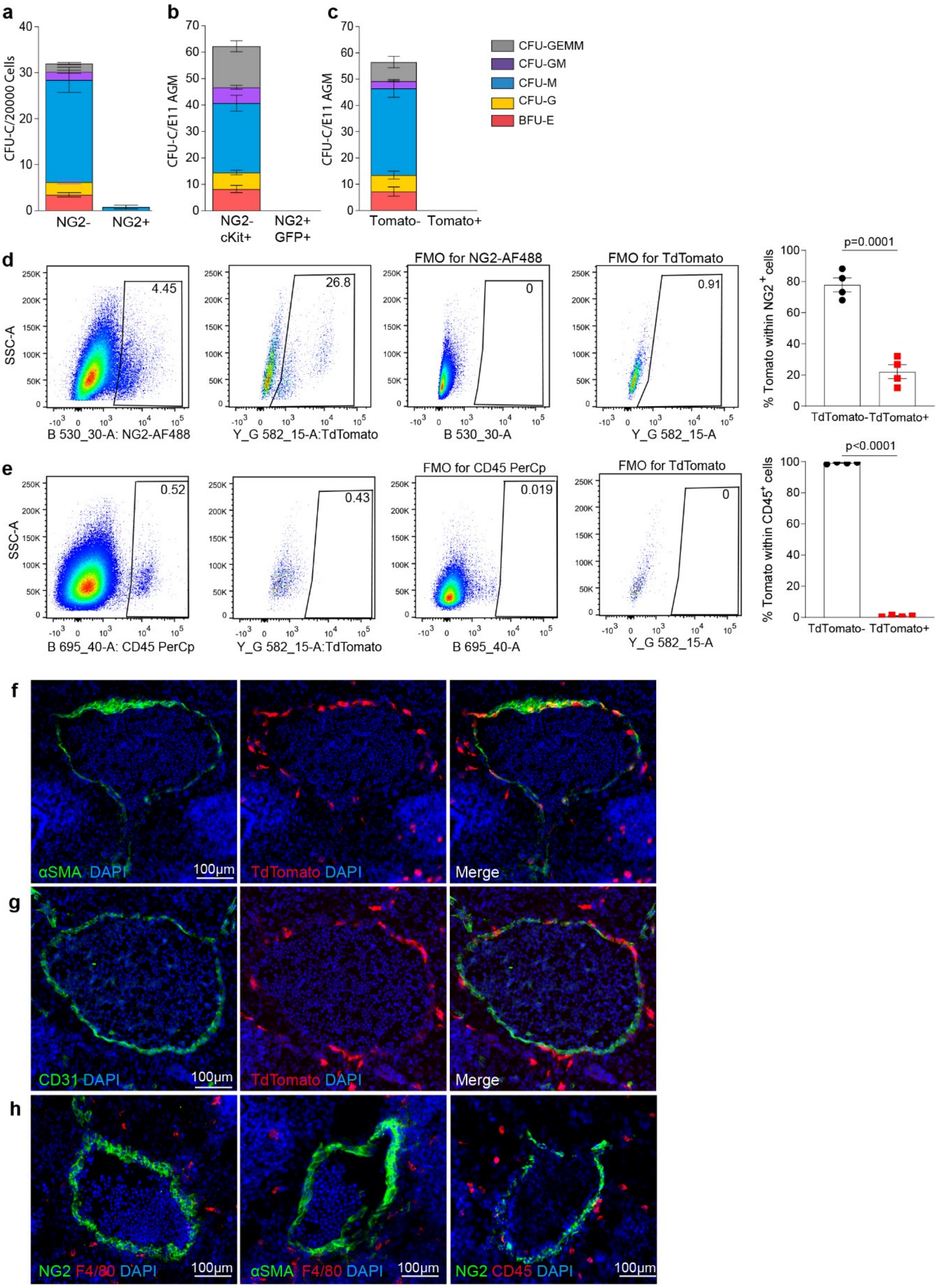

a
CFU-C/20000 Cells
NG2-
NG2+
b
CFU-C/E11 AGM
NG2- cKit+
NG2+ GFP+
c
CFU-C/E11 AGM
Tomato-
Tomato+
CFU-GEMM
CFU-GM
CFU-M
CFU-G
BFU-E
d
SSC-A
B 530_30-A: NG2-AF488
4.45
Y_G 582_15-A:TdTomato
26.8
FMO for NG2-AF488
B 530_30-A
0
FMO for TdTomato
Y_G 582_15-A
0.91
% Tomato within NG2+ cells
p=0.0001
TdTomato-
TdTomato+
e
SSC-A
B 695_40-A: CD45 PerCp
0.52
Y_G 582_15-A:TdTomato
0.43
FMO for CD45 PerCp
B 695_40-A
0.019
FMO for TdTomato
Y_G 582_15-A
0
% Tomato within CD45+ cells
p<0.0001
TdTomato-
TdTomato+
f
αSMA DAPI
100μm
TdTomato DAPI
Merge
g
CD31 DAPI
100μm
TdTomato DAPI
Merge
h
NG2 F4/80 DAPI
100μm
αSMA F4/80 DAPI
100μm
NG2 CD45 DAPI
100μm

Figure S2. NG2$^+$Runx1$^+$ cells do not contribute to hematopoietic lineages in the AGM. Hematopoietic progenitor assays were performed with **(a)** WT NG2$^{+/-}$ E11 AGM cells (n=9, p<0.0001 by Mann-Whitney test, two-tailed), **(b)** NG2$^+$Runx1(GFP)$^+$/NG2$^-$ckit$^+$ cells isolated from Runx1-IRES-GFP E11 AGM (n=4) and **(c)** NG2-Cre;tdTomato$^{+/-}$ cells isolated from E11 AGM (n=4) (Table S5). Representative flow cytometric analysis and quantification of TdTomato expression in NG2$^+$cells **(d)** and CD45$^+$cells **(e)** in the NG2-Cre;tdTomato E11 AGM (n=4, two-tailed, unpaired t-test). All data are presented as Mean values+/-SEM. Source data for a, b, c, d and e are provided as a Source Data file. Immunohistochemistry on transversal sections of frozen E11 AGM obtained from NG2-Cre; TdTomato$^+$ mouse embryos stained with anti-RFP **(f, g,** red**)**, anti-αSMA **(f,** green**)** and CD31 (**g,** green) (n=2, N=2). **h.** Immunohistochemistry on transversal sections of frozen E11 WT AGM stained with NG2 or αSMA (green) and F4/80 (n=1, N=1) or CD45 (red) (n=2, N=2). N = number of independent experiments; n=biological samples.

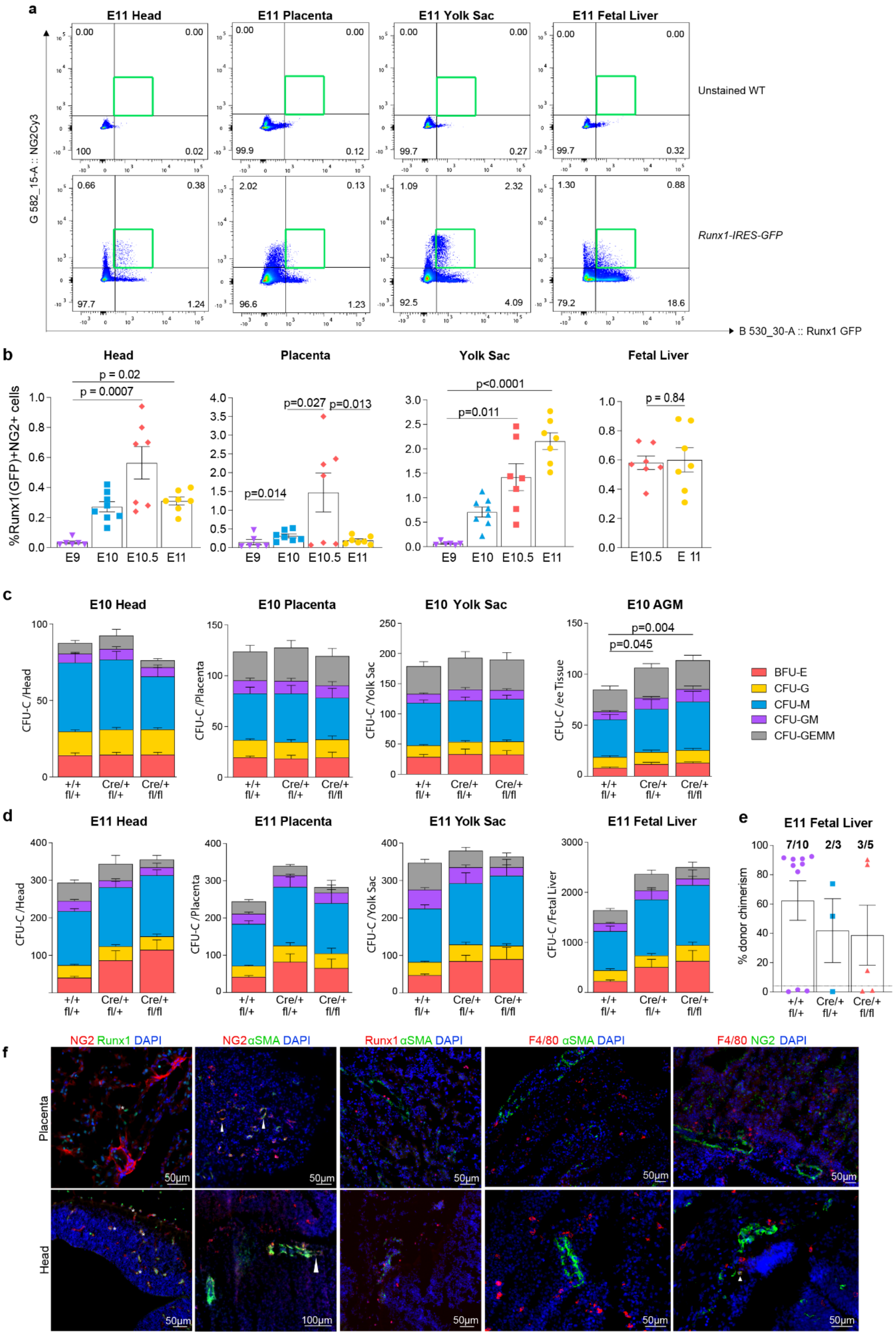
a
E11 Head
E11 Placenta
E11 Yolk Sac
E11 Fetal Liver
Unstained WT
Runx1-IRES-GFP
G 582_15-A :: NG2Cy3
B 530_30-A :: Runx1 GFP
b
Head
Placenta
Yolk Sac
Fetal Liver
%Runx1(GFP)+NG2+ cells
p = 0.02
p = 0.0007
p=0.027
p=0.013
p=0.014
p<0.0001
p=0.011
p = 0.84
E9
E10
E10.5
E11
E 11
c
E10 Head
E10 Placenta
E10 Yolk Sac
E10 AGM
CFU-C /Head
CFU-C /Placenta
CFU-C /Yolk Sac
CFU-C /ee Tissue
p=0.004
p=0.045
+/+ fl/+
Cre/+ fl/+
Cre/+ fl/fl
BFU-E
CFU-G
CFU-M
CFU-GM
CFU-GEMM
d
E11 Head
E11 Placenta
E11 Yolk Sac
E11 Fetal Liver
CFU-C /Fetal Liver
e
E11 Fetal Liver
7/10
2/3
3/5
% donor chimerism
f
NG2 Runx1 DAPI
NG2 αSMA DAPI
Runx1 αSMA DAPI
F4/80 αSMA DAPI
F4/80 NG2 DAPI
Placenta
Head
50μm
100μm

Figure S3. Deletion of *Runx1* in NG2$^+$cells affects selective hematopoietic progenitor types in non-AGM embryonic organs. a. Representative examples of flow cytometric analysis of NG2$^+$Runx1(GFP)$^+$ (green box) in E11 *Runx1-IRES-GFP* hematopoietic organs. E11 WT unstained controls are shown for each tissue. **b.** Percentages of NG2$^+$Runx1(GFP)$^+$ cells in E9/E10/E10.5/E11 Runx1-IRES-GFP hematopoietic organs (n=6/7/7/7 embryos, N=4 independent experiments), determined by flow cytometry. Data are mean ± SEM; Head/YS: Kruskal-Wallis with Dunn's *post-hoc* test, Placenta: One-way ANOVA with Dunn's *post-hoc* test and FL: Unpaired T-Test, Two-tailed. CFU-C numbers per WT, HET and cKO **(c)** E10 and **(d)** E11 head, placenta, yolk-sac, AGM or fetal liver. E10: WT/HET/cKO = 13/8/7 (Table S6) and E11: WT/HET/cKO = 15/9/5 for head, Pl, YS and 15/6/4 for FL (Table S7) **e.** Percentages of donor cell chimerism 4-months post transplantation of E11 WT (n=10), HET (n=3), and cKO (n=5) fetal liver into sub-lethally adult irradiated recipients (1x FL cells transplanted/recipient; N=4). Each dot represents one recipient. Mice are reconstituted when ≥5% donor cells are found in the host peripheral blood (dashed line)**.** WT *($NG2^{+/+}$;$Runx1^{fl/+}$*or *$NG2^{+/+}$;$Runx1^{fl/fl}$),* HET (*NG2-Cre;$Runx1^{fl/+}$*) and cKO AGM (*NG2-Cre;$Runx1^{fl/fl}$*). **f.** Immunohistochemistry on transversal sections of frozen E10 or E11 WT placenta (upper line) and head (bottom line) stained with antibodies against NG2 (placenta/head n= 4/2), Runx1 (n=4/1), αSMA (n=4/2), and F4/80 (n=1/1). DAPI shows nuclei (blue). N=4. All data are presented as Mean values+/-SEM. N = number of independent experiments; n = number of biological samples. Source data for b, c, d and e are provided as a Source Data file.

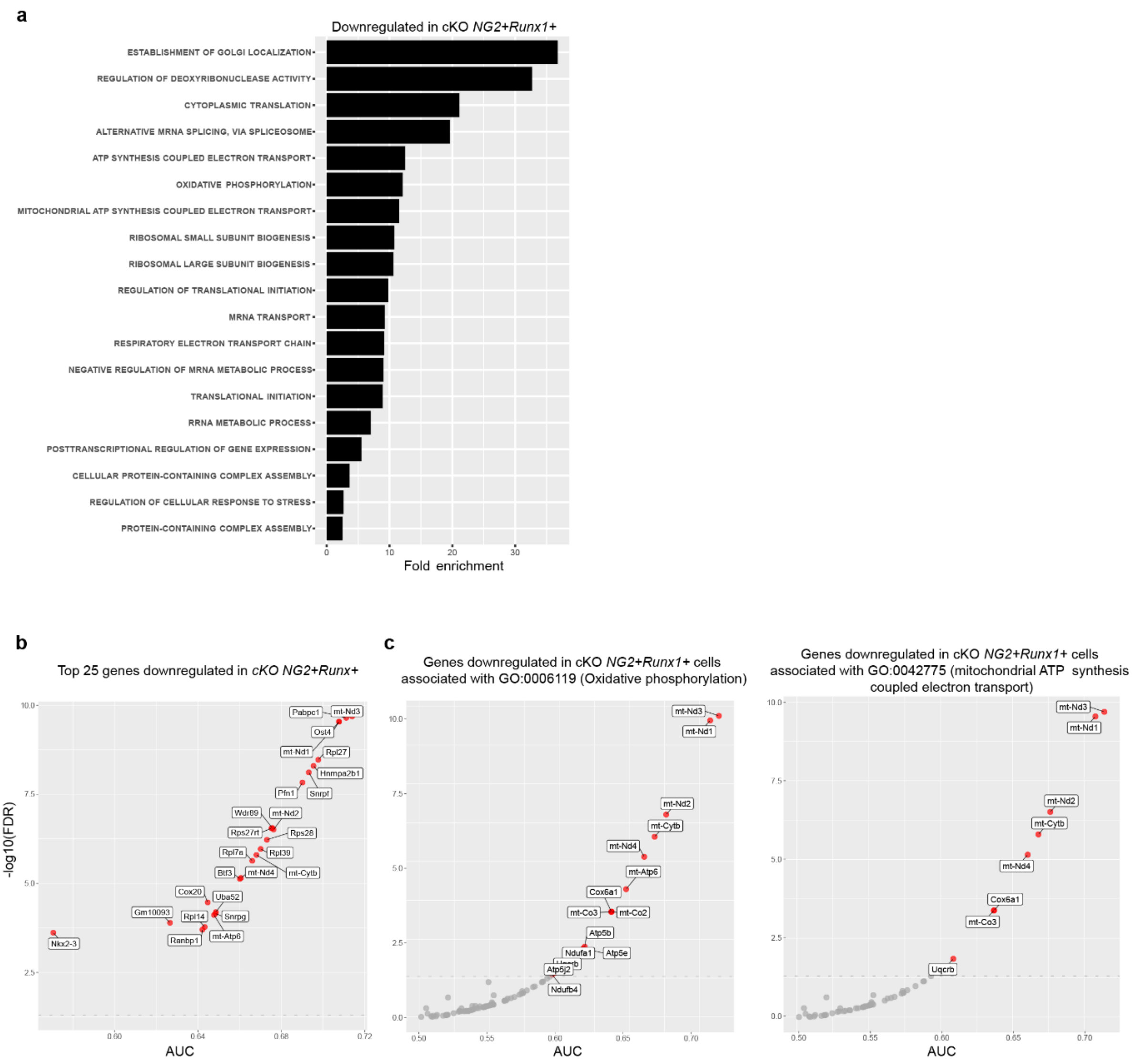

Figure S4. scRNA-seq comparison between *NG2*$^{+}$*Runx1*$^{+}$ WT and cKO cells. a. Barplot of fold enrichment for selected GO biological processes significantly overrepresented in genes significantly downregulated in *NG2*$^{+}$*Runx1*$^{+}$*Acta2*$^{+}$ cKO AGM. Scatter plots of AUC vs –log10(FDR) showing the top 25 downregulated genes in cKO NG2+Runx1+ cells **(b)** or associated with selected GO terms 0006119 and 0042775 **(c).** Red dots represent significantly downregulated genes (FDR<0.05); dashed line shows FDR=0.05**.**

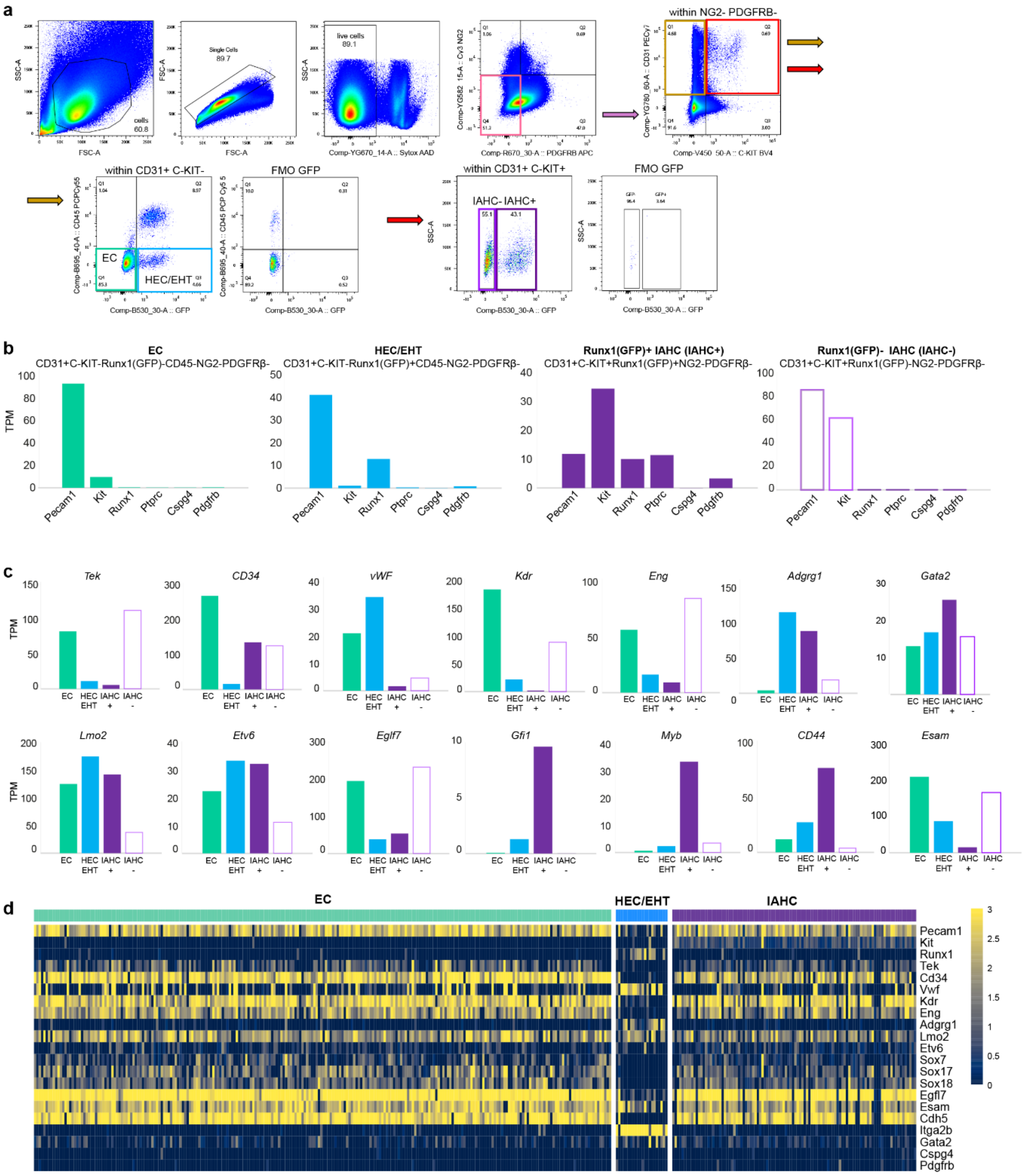
a
within NG2- PDGFRB-
within CD31+ C-KIT-
FMO GFP
within CD31+ C-KIT+
IAHC- IAHC+
FMO GFP
EC
HEC/EHT
b
EC
CD31+C-KIT-Runx1(GFP)-CD45-NG2-PDGFRβ-
HEC/EHT
CD31+C-KIT-Runx1(GFP)+CD45-NG2-PDGFRβ-
Runx1(GFP)+ IAHC (IAHC+)
CD31+C-KIT+Runx1(GFP)+NG2-PDGFRβ-
Runx1(GFP)- IAHC (IAHC-)
CD31+C-KIT+Runx1(GFP)-NG2-PDGFRβ-
TPM
Pecam1
Kit
Runx1
Ptprc
Cspg4
Pdgfrb
c
Tek
CD34
vWF
Kdr
Eng
Adgrg1
Gata2
Lmo2
Etv6
Eglf7
Gfi1
Myb
CD44
Esam
EC
HEC EHT
IAHC +
IAHC -
d
EC
HEC/EHT
IAHC
Pecam1
Kit
Runx1
Tek
Cd34
Vwf
Kdr
Eng
Adgrg1
Lmo2
Etv6
Sox7
Sox17
Sox18
Egfl7
Esam
Cdh5
Itga2b
Gata2
Cspg4
Pdgfrb

Figure S5: WT IAHCs are composed of both Runx1^{+} and Runx1^{-} cells a. ECs (CD31^{+}ckit^{-}CD45^{-}Runx1^{-}, yellow then green gate), HECs (CD31^{+}ckit^{-}CD45^{-}Runx1^{+}, yellow then blue gate), and IAHC cells (CD31^{+}ckit^{+}Runx1^{+} and Runx1^{-}, red gate then purple gates) were purified by FACS from pooled PDGFRβ^{-}NG2^{-} Runx1-GFP/GFP and Runx1-GFP/+ E11 AGM live cells (Sytox^{-}). **b.** Post-sort purity check by bulk RNA sequencing was performed on all four cell populations isolated. **c.** The expression of selected genes from scRNA-seq (a) are shown in the bulk RNA-seq data in all four sorted cell populations ECs (green bars), HEC/EHT (blue bars), Runx1^{+} IAHCs (full purple bars) and Runx1^{-} IAHCs (empty purple bars). TPM: Transcript per Million mapped reads values. **d.** Heatmap showing the expression of selected genes that characterise EC (*Pecam1^{+}Kit^{-}Runx1^{-}),* HEC/HET (*Pecam1^{+}Kit^{-}Runx1^{+}),* and IAHC clusters (*Pecam1^{+}Kit^{+})* at single cell level including those that contributed to their identification.

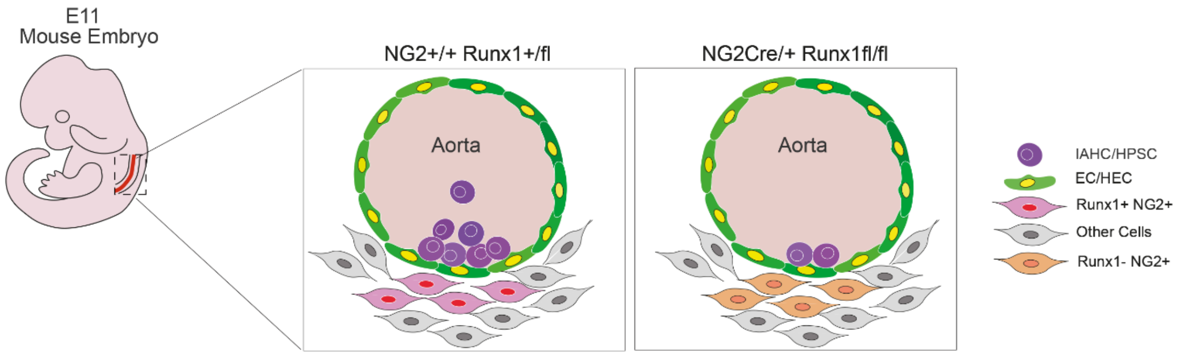

Figure S6. Schematic showing the functional role of NG2$^+$Runx1$^+$ PC/vSMCs in the E11 AGM hematopoietic niche in the mouse embryo *in vivo*. In the WT (*NG2*$^{+/+}$*Runx1*$^{+/fl}$) E11 AGM, the presence of NG2$^+$Runx1$^+$ PC/vSMCs mediates the generation/maintenance of intra-aortic hematopoietic clusters (IAHCs) including functional hematopoietic progenitor/stem cells (HSPCs). Conditional deletion of Runx1 in NG2$^+$ (*NG2*$^{Cre/+}$*Runx1*$^{fl/fl}$) cells leads to significant transcriptomic changes in PC/vSMCs, ECs and IAHCs and dramatic reduction in the numbers of IAHCs and functional HSPCs.

Table S1. Antibodies tested on wholemount embryos (Figures 1 a, b, e and g) on WT (+;Runx1 $^{fl/+}$ or +;Runx1 $^{fl/fl}$) and cKO (NG2Cre;Runx1$^{fl/fl}$).

Antibody tested by wholemount embryo staining	WT stage and ID	cKO stage and ID
**αSMA**	9<br>E11 litter 19: 4, 5, 6<br>E10.5. litter 37: 1, 5, 6, 7, 8, 10	7<br>E11 litter 19: 1, 8, 7<br>E10.5 litter 41: 3, 5, 9, 10
**CD31**	10<br>E11 litter 19: 4, 5, 6<br>E10.5 litter 37: 1, 3, 5, 6, 7, 8, 10	7<br>E11 litter 19: 1, 8, 7<br>E10.5 litter 41: 3, 5, 9, 10
**cKit**	3<br>E11 litter 19: 4,<br>E10.5 litter 37: 1, 10	2<br>E11 litter 19: 1,<br>E10.5 litter 41: 5
**Runx1**	4<br>E10.5 litter 37: 3, 5, 7<br>E10.5 litter 69: 4	
**NG2**	3<br>E10.5 litter 37: 6,8<br>E10.5 litter 69: 4	1<br>E10.5 litter 41: 9
Total number of embryos **(n)**	11	7
Total number of experiments **(N)**	6	4
Total number of these antibody combinations	3	3

Table S2. Summary of CFU-C data obtained from WT, HET and cKO E10.5/E11 AGM and statistical analyses performed.

				Number of embryos			Average			Fold decrease							Multiple comparisons		
Tissue	Day	Somite pairs	CFU-type	WT	HET	KO	WT	HET	KO	WT vs HET	WT vs KO	HET vs KO	Statistical test	p-value		Post-hoc	WT vs HET	WT vs KO	Het vs KO
AGM	10.5	31-38	BFU-E	14	10	5	21.79	22.30	6.40	0.98	3.40	3.48	1-Way ANOVA	0.0006	***	Tukey's	ns	0.0009	0.0011
			CFU-G				18.21	19.70	2.80	0.92	6.50	7.04	1-Way ANOVA	0.0004	***	Tukey's	ns	0.0008	0.0005
			CFU-M				29.36	37.70	6.80	0.78	4.32	5.54	1-Way ANOVA	0.0045	**	Tukey's	ns	0.0253	0.0033
			CFU-GM				14.50	16.40	0.00	0.88	-	-	Kruskal-Wallis	0.0022	**	Dunn's	ns	0.0049	0.0030
			CFU-GEMM				13.71	14.70	1.60	0.93	8.57	9.19	Kruskal-Wallis	0.0060	**	Dunn's	ns	0.0114	0.0082
			Total				97.57	110.80	17.60	0.88	5.54	6.30	1-Way ANOVA	<0.0001	****	Tukey's	ns	0.0002	<0.0001
AGM	11	43-52	BFU-E	22	8	19	27.5	24.25	6.6	1.13	4.17	3.67	1-way ANOVA	<0.0001	****	Tukey's	ns	<0.0001	0.0001
			CFU-G				41.5	40.0	14.7	1.04	2.82	2.72	Kruskal-Wallis	<0.0001	****	Dunn's	ns	<0.0001	0.0006
			CFU-M				76.1	30.75	20.4	2.47	3.73	1.51	Kruskal-Wallis	<0.0001	***	Dunn's	0.0020	<0.0001	ns
			CFU-GM				22.04	15.75	2.36	1.40	9.34	6.67	Kruskal-Wallis	<0.0001	****	Dunn's	ns	<0.0001	0.0040
			CFU-GEMM				28.22	24.62	6.78	1.15	4.16	3.63	Kruskal-Wallis	<0.0001	****	Dunn's	ns	<0.0001	0.0009
			Total				195.27	135.37	53.26	1.13	3.67	3.26	1-way ANOVA	<0.0001	****	Tukey's	ns	<0.0001	<0.0001

Table S3. Summary of reconstitution efficiency data obtained from WT, HET and cKO E11 AGM, adult bone marrow, and fetal liver with statistical analyses performed.

		Total mice (Tm)	Reconstituted mice (Rm)	Proportion of reconstitution (Rm)/(Tm)	Proportion comparisons	Statistical test	p-value		z-value
Figure 1k	WT	6	4	0.67	WT vs HET	One-tailed Z Score test for 2 population proportions	0.025	*	1.958
	HET	7	1	0.14	WT vs KO	One-tailed Z Score test for 2 population proportions	0.040	*	1.755
	KO	6	1	0.17	HET vs KO	One-tailed Z Score test for 2 population proportions	0.440	ns	1.415
Figure 7f	WT	29	18	0.62	WT vs HET	One-tailed Z Score test for 2 population proportions	0.024	*	1.979
	HET	11	3	0.27	WT vs KO	One-tailed Z Score test for 2 population proportions	0.002	**	2.905
	KO	20	4	0.20	HET vs KO	One-tailed Z Score test for 2 population proportions	0.326	ns	0.447
Figure 7h	WT	9	6	0.67	WT vs HET	One-tailed Z Score test for 2 population proportions	0.386	ns	-0.289
	HET	4	3	0.75	WT vs KO	One-tailed Z Score test for 2 population proportions	0.074	ns	1.442
	KO	9	3	0.33	HET vs KO	One-tailed Z Score test for 2 population proportions	0.081	ns	1.402
Figure S3, e	WT	10	7	0.7	WT vs HET	One-tailed Z Score test for 2 population proportions	0.460	ns	0.099
	HET	3	2	0.67	WT vs KO	One-tailed Z Score test for 2 population proportions	0.348	ns	0.387
	KO	5	3	0.6	HET vs KO	One-tailed Z Score test for 2 population proportions	0.421	ns	0.198

Table S4. Summary of percentage of reconstitution data obtained from WT, HET and cKO E11 AGM, adult bone marrow, and fetal liver with statistical analyses performed.

		Total mice	% of reconstitution (Average)		Fold change	Statistical test	p-value		Post-hoc	Multiple comparisons		
										WT vs HET	WT vs KO	Het vs KO
Figure 1k	WT	6	23.63	WT vs HET	1.84	Kruskal-Wallis	0.222	ns	Dunn's	ns	ns	ns
	HET	7	12.83	WT vs KO	2.49							
	KO	6	9.5	HET vs KO	1.35							
Figure 7f	WT	29	33.39	WT vs HET	2.12	Kruskal-Wallis	0.0027	**	Dunn's	ns	** 0.0019	ns
	HET	11	15.73	WT vs KO	3.70							
	KO	20	9.03	HET vs KO	1.74							
Figure 7h	WT	9	26.83	WT vs HET	1.68	Kruskal-Wallis	0.321	ns	Dunn's	ns	ns	ns
	HET	4	31	WT vs KO	2.12							
	KO	9	12.68	HET vs KO	2.44							
Figure S3, e	WT	10	62.34	WT vs HET	1.49	Kruskal-Wallis	0.331	ns	Dunn's	ns	ns	ns
	HET	3	41.87	WT vs KO	1.61							
	KO	5	38.72	HET vs KO	1.08							

Table S5. Summary of CFU-C data obtained from E11 AGM sorted cells and statistical analyses performed.

				Average					
Tissue (number of embryos)	Day	Somite pairs	CFU-type	NG2-	NG2+	Statistical test	p-value		Post-hoc
C57BL6 AGM (n=9)	11	43-52	BFU-E	3.44	0	Two-tailed Mann-Whitney test	<0.0001	****	-
			CFU-G	2.77	0	Two-tailed Mann-Whitney test	<0.0001	****	-
			CFU-M	22.22	0.77	Two-tailed Mann-Whitney test	<0.0001	****	-
			CFU-GM	1.77	0	Two-tailed Mann-Whitney test	<0.0001	****	-
			CFU-GEMM	1.77	0	Two-tailed Mann-Whitney test	0.0004	***	-
			Total	32.44	0.77	Two-tailed Mann-Whitney test	<0.0001	****	-
Runx1-IRES-GFP AGM (n=4)	11	43-52	CFU-type	NG2- cKit+	Runx1(GFP)+ NG2+	Statistical test	p-value		Post-hoc
			BFU-E	8.25	0	-	-	-	-
			CFU-G	6.25	0	-	-	-	-
			CFU-M	26.25	0	-	-	-	-
			CFU-GM	6.00	0	-	-	-	-
			CFU-GEMM	15.5	0	-	-	-	-
			Total	62.75	0	-	-	-	-
NG2-Cre TdTomato+ AGM (n=4)	11	43-52	CFU-type	Tomato-	Tomato+	Statistical test	p-value		Post-hoc
			BFU-E	7.25	0	-	-	-	-
			CFU-G	6	0	-	-	-	-
			CFU-M	33	0	-	-	-	-
			CFU-GM	2.75	0	-	-	-	-
			CFU-GEMM	7.25	0	-	-	-	-
			Total	56.25	0	-	-	-	-

Table S6. Summary of CFU-C data obtained from WT, HET and cKO E10 hematopoietic organs and statistical analyses performed.

				Number of embryos			Average			Fold decrease							Multiple comparisons		
Tissue	Day	Somite pairs	CFU-type	WT	HET	KO	WT	HET	KO	WT vs HET	WT vs KO	HET vs KO	Statistical test	p-value		Post-hoc	WT vs HET	WT vs KO	Het vs KO
AGM	10	29-31	BFU-E	13	8	7	8.38	12.00	13.14	0.70	0.64	0.91	1-way ANOVA	0.0100	**	Tukey's	ns	0.0149	ns
			CFU-G				10.54	11.75	12.57	0.90	0.84	0.93	Kruskal-Wallis	0.1819	ns	Dunn's	ns	ns	ns
			CFU-M				36.92	42.25	47.43	0.87	0.78	0.89	Kruskal-Wallis	0.5962	ns	Dunn's	ns	ns	ns
			CFU-GM				7.62	10.88	12.29	0.70	0.62	0.89	Kruskal-Wallis	0.1380	ns	Dunn's	ns	ns	ns
			CFU-GEMM				21.46	29.50	28.43	0.73	0.75	1.04	1-way ANOVA	0.2852	ns	Tukey's	ns	ns	ns
			Total				84.92	106.88	113.86	0.79	0.75	0.94	Kruskal-Wallis	0.0023	**	Dunn's	0.0455	0.0040	ns
Head	10	29-31	BFU-E	13	8	7	14.15	14.50	14.57	0.98	0.97	1.00	Kruskal-Wallis	0.9908	ns	Dunn's	ns	ns	ns
			CFU-G				15.54	16.50	16.57	0.94	0.94	1.00	1-way ANOVA	0.7636	ns	Tukey's	ns	ns	ns
			CFU-M				45.08	45.75	34.57	0.99	1.30	1.32	Kruskal-Wallis	0.3696	ns	Dunn's	ns	ns	ns
			CFU-GM				5.85	7.00	6.00	0.84	0.97	1.17	Kruskal-Wallis	0.7055	ns	Dunn's	ns	ns	ns
			CFU-GEMM				7.00	8.75	4.57	0.80	1.53	1.91	Kruskal-Wallis	0.7107	ns	Dunn's	ns	ns	ns
			Total				88.00	92.75	81.71	0.95	1.08	1.14	1-way ANOVA	0.7761	ns	Tukey's	ns	ns	ns
PL	10	29-31	BFU-E	13	8	7	19.69	18.50	19.43	1.06	1.01	0.95	1-way ANOVA	0.955	ns	Tukey's	ns	ns	ns
			CFU-G				17.08	16.25	18.00	1.05	0.95	0.90	Kruskal-Wallis	0.4923	ns	Dunn's	ns	ns	ns
			CFU-M				45.85	47.75	40.86	0.96	1.12	1.17	Kruskal-Wallis	0.6755	ns	Dunn's	ns	ns	ns
			CFU-GM				12.77	12.50	12.29	1.02	1.04	1.02	Kruskal-Wallis	0.9125	ns	Dunn's	ns	ns	ns
			CFU-GEMM				28.46	32.75	28.86	0.87	0.99	1.13	Kruskal-Wallis	0.9087	ns	Dunn's	ns	ns	ns
			Total				123.85	127.75	119.43	0.97	1.04	1.07	Kruskal-Wallis	0.9725	ns	Dunn's	ns	ns	ns
YS	10	29-31	BFU-E	13	8	7	28.77	33.20	32.67	0.87	0.88	0.99	Kruskal-Wallis	0.7485	ns	Dunn's	ns	ns	ns
			CFU-G				19.23	20.50	21.71	0.94	0.89	0.94	1-way ANOVA	0.5164	ns	Tukey's	ns	ns	ns
			CFU-M				70.31	68.50	70.29	1.03	1.00	0.97	1-way ANOVA	0.9649	ns	Tukey's	ns	ns	ns
			CFU-GM				14.92	18.50	14.86	0.81	1.00	1.24	Kruskal-Wallis	0.4754	ns	Dunn's	ns	ns	ns
			CFU-GEMM				45.85	52.25	50.57	0.88	0.91	1.03	Kruskal-Wallis	0.7930	ns	Dunn's	ns	ns	ns

			Total				179.08	187.00	187.71	0.96	0.95	179.08	1-way ANOVA	0.4144	ns	Tukey’s	ns	ns	ns

Table S7. Summary of CFU-C data obtained from WT, HET and cKO E11 hematopoietic organs and statistical analyses performed.

				Number of embryos			Average			Fold decrease							Multiple comparisons		
Tissue	Day	Somite pairs	CFU-type	WT	HET	KO	WT	HET	KO	WT vs HET	WT vs KO	HET vs KO	Statistical test	p-value		Post-hoc	WT vs HET	WT vs KO	Het vs KO
Head	11	43-52	BFU-E	15	9	5	40.40	86.78	114.80	0.47	0.35	0.76	Kruskal-Wallis	0.0145	*	Dunn's	ns	0.0117	ns
			CFU-G				33.60	37.67	36.00	0.89	0.93	1.05	1-way ANOVA	0.6542	ns	Tukey's	ns	ns	ns
			CFU-M				144.00	157.11	162.60	0.92	0.89	0.97	1-way ANOVA	0.4887	ns	Tukey's	ns	ns	ns
			CFU-GM				27.40	18.22	21.20	1.50	1.29	0.86	Kruskal-Wallis	0.2604	ns	Dunn's	ns	ns	ns
			CFU-GEMM				48.20	44.11	21.00	1.09	2.30	2.10	Kruskal-Wallis	0.0928	ns	Dunn's	ns	ns	ns
			Total				293.60	343.89	355.60	0.85	0.83	0.97	1-way ANOVA	0.1886	ns	Tukey's	ns	ns	ns
FL	11	43-52	BFU-E	15	6	4	224.62	506.67	630.00	0.44	0.36	0.80	Kruskal-Wallis	0.0707	ns	Dunn's	ns	ns	ns
			CFU-G				218.46	233.33	315.00	0.94	0.69	0.74	Kruskal-Wallis	2833	ns	Dunn's	ns	ns	ns
			CFU-M				783.08	1120.00	1205.00	0.70	0.65	0.93	1-way ANOVA	0.1799	ns	Tukey's	ns	ns	ns
			CFU-GM				158.46	180.00	125.00	0.88	1.27	1.44	1-way ANOVA	1.022	ns	Tukey's	ns	ns	ns
			CFU-GEMM				204.62	326.67	230.00	0.63	0.89	1.42	1-way ANOVA	0.3058	ns	Tukey's	ns	ns	ns
			Total				1646.15	2366.67	2505.00	0.70	0.66	0.94	Kruskal-Wallis	0.3386	ns	Dunn's	ns	ns	ns
PL	11	43-52	BFU-E	15	9	5	42.00	82.33	65.80	0.51	0.64	1.25	Kruskal-Wallis	0.1279	ns	Dunn's	ns	ns	ns
			CFU-G				29.60	43.89	39.40	0.67	0.75	1.11	Kruskal-Wallis	0.128	ns	Dunn's	ns	ns	ns
			CFU-M				142.40	163.44	187.00	0.87	0.76	0.87	Kruskal-Wallis	0.1930	ns	Dunn's	ns	ns	ns
			CFU-GM				27.60	30.56	27.80	0.90	0.99	1.10	1-way ANOVA	0.1111	ns	Tukey's	ns	ns	ns
			CFU-GEMM				33	26	15.2	1.27	2.17	1.71	1 -way ANOVA	0.0899	ns	Tukey's	ns	ns	ns
			Total				243.80	340.56	283.00	0.72	0.86	1.20	Kruskal-Wallis	0.2299	ns	Dunn's	ns	ns	ns
YS	11	43-52	BFU-E	15	9	5	47.40	84.89	90.20	0.56	0.53	0.94	1-way ANOVA	0.0387	*	Tukey's	ns	ns	ns
			CFU-G				35.20	44.44	36.00	0.79	0.98	1.23	1-way ANOVA	0.1909	ns	Tukey's	ns	ns	ns
			CFU-M				265.12	195.9	210.62	1.35	1.26	0.93	1-way ANOVA	0.4192	ns	Tukey's	ns	ns	ns
			CFU-GM				50.80	42.56	22.60	1.19	2.25	1.88	1-way ANOVA	0.021	*	Tukey's	ns	0.0159	ns
			CFU-GEMM				71.60	44.44	28.60	1.61	2.50	1.55	Kruskal-Wallis	0.0272	*	Dunn's	ns	0.0389	ns

			Total				347.40	399.17	364.40	0.87	0.95	1.10	1-way ANOVA	0.5799	ns	Tukey's	ns	ns	ns

Table S8. Summary of CFU-C data obtained from WT, HET and cKO adult bone marrow and statistical analyses performed.

			Number of adult mice			Average			Fold decrease							Multiple comparisons		
Tissue	Stage	CFU-type	WT	HET	KO	WT	HET	KO	WT vs HET	WT vs KO	HET vs KO	Statistical test	p-value		Post-hoc	WT vs HET	WT vs KO	Het vs KO
Bone Marrow	Adult	BFU-E	13	7	8	6.86	4.38	7.33	1.56	0.94	0.60	1-way ANOVA	0.1888	ns	Tukey's	ns	ns	ns
		CFU-G				9.54	6.76	8.63	1.41	1.11	0.78	1-way ANOVA	0.2758	ns	Tukey's	ns	ns	ns
		CFU-M				18.18	15.86	24.58	1.15	0.74	0.65	1-way ANOVA	0.1514	ns	Tukey's	ns	ns	ns
		CFU-GM				11.85	11.38	15.33	1.04	0.77	0.74	1-way ANOVA	0.4001	ns	Tukey's	ns	ns	ns
		CFU-GEMM				16.82	9.33	12.54	1.80	1.34	0.74	Kruskal-Wallis	0.0523	ns	Dunn's	ns	ns	ns
		Total				63.14	46.95	68.04	1.34	0.93	0.69	1-way ANOVA	0.2248	ns	Tukey's	ns	ns	ns
