# Supplementary material for: Runx1+ vascular smooth muscle cells are essential for hematopoietic stem and progenitor cell development in vivo
Source: Nat Commun. 2024 Feb 23;15:1653. doi: 10.1038/s41467-024-44913-z (PMC10891074; doi:10.1038/s41467-024-44913-z)
Supplement: Supplementary file 2 — Reporting Summary [file 41467_2024_44913_MOESM2_ESM.pdf]

nature portfolio

Corresponding author(s): Mihaela Crisan

Last updated by author(s): 8 December 2023

Reporting Summary

Nature Portfolio wishes to improve the reproducibility of the work that we publish. This form provides structure for consistency and transparency in reporting. For further information on Nature Portfolio policies, see our Editorial Policies and the Editorial Policy Checklist.

Statistics

For all statistical analyses, confirm that the following items are present in the figure legend, table legend, main text, or Methods section.

n/a	Confirmed	
☐	☒	The exact sample size (*n*) for each experimental group/condition, given as a discrete number and unit of measurement
☐	☒	A statement on whether measurements were taken from distinct samples or whether the same sample was measured repeatedly
☐	☒	The statistical test(s) used AND whether they are one- or two-sided<br>*Only common tests should be described solely by name; describe more complex techniques in the Methods section.*
☒	☐	A description of all covariates tested
☐	☒	A description of any assumptions or corrections, such as tests of normality and adjustment for multiple comparisons
☐	☒	A full description of the statistical parameters including central tendency (e.g. means) or other basic estimates (e.g. regression coefficient) AND variation (e.g. standard deviation) or associated estimates of uncertainty (e.g. confidence intervals)
☐	☒	For null hypothesis testing, the test statistic (e.g. *F*, *t*, *r*) with confidence intervals, effect sizes, degrees of freedom and *P* value noted<br>*Give P values as exact values whenever suitable.*
☒	☐	For Bayesian analysis, information on the choice of priors and Markov chain Monte Carlo settings
☒	☐	For hierarchical and complex designs, identification of the appropriate level for tests and full reporting of outcomes
☐	☒	Estimates of effect sizes (e.g. Cohen's *d*, Pearson's *r*), indicating how they were calculated

Our web collection on statistics for biologists contains articles on many of the points above.

Software and code

Policy information about availability of computer code

Data collection	Flow cytometry and cell sorting data collection was performed with BD LSR Fortessa 4 laser (BD Biosciences), BD FACS ARIA Fusion with BD FACS Diva Software V8.0.l or Novocyte Flow Cytometer (ACEA Biosciences) and NovoExpressTM Software (1.5.0). Confocal (3D whole mount) images were taken using a a Leica SP8 confocal microscope with the Leica Application Suite X software (v3.5.5.19976). lmmunohistochemistry pictures were taken using an inverted widefield fluorescence microscope (Zeiss Observer) with the Zen 2.3 pro (blue edition) software, Illumina Novaseq 6000 S1 lane, 10X Genomics Cell Ranger (v3.l.0), Smarter 2 and Illumina HiSeq2500 for bulk RNA-seq.
Data analysis	Flow cytometry and sort data were analysed with FlowJo X vl0.0.7. All statistical analyses and graphs were made using GraphPad Prism 7. Stitching and deconvolution were done using Huygens Pro v19.10 and images were analysed with FIJl/lmageJ 1.52e and Zen Blue Sofware. l0X Genomics Cell Ranger (v3.1.0), Cell Ranger 'count' (v3.1.0), AmiGO (v.2.5.13); R/Bioconductor packages: DropletUtils (v.1.10.3), scater (v.1.14.6), batchelor (v.1.2.4), scran (v.1.18.7), nichenetr (v.1.0.0), slingshot (v.2.2.1), HiSat2 (version 2.0.4), Bioconductor Genome Alignments Package (vl.8.1).

For manuscripts utilizing custom algorithms or software that are central to the research but not yet described in published literature, software must be made available to editors and reviewers. We strongly encourage code deposition in a community repository (e.g. GitHub). See the Nature Portfolio guidelines for submitting code & software for further information.

Data

Policy information about availability of data

All manuscripts must include a data availability statement. This statement should provide the following information, where applicable:

- Accession codes, unique identifiers, or web links for publicly available datasets
- A description of any restrictions on data availability
- For clinical datasets or third party data, please ensure that the statement adheres to our policy

The scRNA-seq data and the bulk RNA-seq data from this study are publicly available on the Gene Expression Omnibus (GEO) database https://www.ncbi.nlm.nih.gov/geo/, accession numbers GSE178981 https://www.ncbi.nlm.nih.gov/geo/query/acc.cgi?acc=GSE178981 and GSE229850 respectively https://www.ncbi.nlm.nih.gov/geo/query/acc.cgi?acc=GSE229850.

Field-specific reporting

Please select the one below that is the best fit for your research. If you are not sure, read the appropriate sections before making your selection.

☒ Life sciences ☐ Behavioural & social sciences ☐ Ecological, evolutionary & environmental sciences

For a reference copy of the document with all sections, see nature.com/documents/nr-reporting-summary-flat.pdf

Life sciences study design

All studies must disclose on these points even when the disclosure is negative.

Sample size	The sample size for this study was determined based on previous published work such as Sa da Bandeira et al, Cell Reports, 2022, Crisan et al, Nature Communications, 2015, Crisan et al, Stem Cell Reports, 2016.
Data exclusions	No data were excluded from the analyses.
Replication	All replication attempts were successful and numbers detailed in each Figure legend. The number of independent experiments performed for Fig 1d, was 5, Fig 1h, N=4, i (N=7), j (N=11) and 1k (N=4). For Figure 7, we performed 7 independent experiments for e and f and 3 experiments for Fig 7h.
Randomization	Samples were allocated in groups based on their genotype (NG2cre/+; Runx1 +/+, fl/+ or fl/fl), based on the cell surface markers used for their identification (PC/vSMCs, Hemogenic/Endothelial Cells or Intra-aortic/Hematopoietic Cells) and based on the transgene expression (Tomato+ versus Tomato- and GFP+ versus GFP-)
Blinding	The scientist who counted the number of haematopoietic progenitors (CFU-C) in all NG2CreRunx1 WT, HET and KO and C57BL6 groups was blinded in regards to the genotype of the samples. A different scientist took the numbers provided, associated them with their respective genotype and analyzed the data. The blinding was not possible with Tomato+/- and GFP+NG2 +/- CFUs when they were counted since the dishes were labeled post FACS sort. However, 2 or 3 scientists checked/counted the dishes for their CFU content.

Reporting for specific materials, systems and methods

We require information from authors about some types of materials, experimental systems and methods used in many studies. Here, indicate whether each material, system or method listed is relevant to your study. If you are not sure if a list item applies to your research, read the appropriate section before selecting a response.

Materials & experimental systems

n/a	Involved in the study	
☐	☒	Antibodies
☒	☐	Eukaryotic cell lines
☒	☐	Palaeontology and archaeology
☐	☒	Animals and other organisms
☒	☐	Human research participants
☒	☐	Clinical data
☒	☐	Dual use research of concern

Methods

n/a	Involved in the study	
☒	☐	ChIP-seq
☐	☒	Flow cytometry
☒	☐	MRI-based neuroimaging

Antibodies

Antibodies used	Immunohistochemistry/wholemount staining: NG2 (Rabbit polyclonal, Millipore, ab5320), NG2 (Rat anti-mouse, R&D systems, MAB6689), Rabbit anti-RFP (Rockland, 600-401-379), CD45 (Goat anti-mouse, R&D systems, AF114), F4/80 (Rat anti-mouse, Abcam, ab6640), Runx1,2,3 (Rabbit anti-mouse, Abcam, ab92336) CD31 (Biotinylated rat anti-mouse, BD Pharmingen, 553371), CD146-AF488

(Rat anti-mouse, Biolegend, 134707), αSMA-Cy3 (Mouse anti-mouse, Sigma, C6198) αSMA-FITC (Mouse anti-mouse, Sigma, F3777), cKit (BD Bioscience, 553352); Goat anti-rabbit Alexa 488 (Invitrogen, A11008), Goat ant-rabbit Alexa 546 (Invitrogen, A11035), Goat anti-rabbit Alexa 647 (Invitrogen, A21244), Goat anti-rat Alexa 594 (Invitrogen, A11007), Donkey anti- Goat Alexa 594 (Invitrogen, A11058) , Chicken anti-rat Alexa 647 (Invitrogen, A21472), Goat anti-rabbit Alexa 594 (Life Technologies, A11012), Streptavidin FITC (BD Pharmingen, 554060), Streptavidin 555 (Invitrogen, S32355) Streptavidin 647 (Life Technologies, S21374), Streptavidin Cy3 (Sigma, S6402).

Flow Cytometry embryonic organs: NG2 Cy3 (Millipore, ab5320c3), NG2 AF488 (Millipore, ab5320a4), CD45 PerCpCy5.5 (BD Pharmingen, 550994), cKit BV421 (BD Horizon, 562609), CD31 PECy7 (eBioscience, 25-0311-82), PDGFRB APC (Biolegend, 136008).

Flow Cytometry BM LSK SLAM: CD4 (BD Biosciences; 553648), CD5 (BD Biosciences, 553018), CD8a (BD Biosciences, 553028), CD11b/Mac-1 (BD Biosciences, 553309), CD45R/B220 (BD Biosciences, 553086), Gr-1/Ly-6G/C (BD Biosciences, 553125), Ter119 (BD Biosciences, 553672), Streptavidin PerCp (BioLegend, 405213), cKit APC (Biolegend, 105812), Sca-1 APC Cy7 (Biolegend, 122514), CD48 PE (Biolegend, 103406), CD150 PE Cy7 (Biolegend, 115914).

Flow cytometry transplantation (Biolegend): CD45.1-FITC (110706), CD45.2-Pacific Blue (109802), CD4 (130310), CD8a (100708), CD11b/Mac-1 APC (101212), CD19 (APC Cy7 (115530), Gr-1/Ly-6G/C PE Cy7 (108416).

Validation

Primary antibodies used in this study were validated in previous published work, in similar mouse tissues, by us and others including Sa da Bandeira et al, Cell Reports, 2022, Murray, Gonzalez et al, Nature Communications, 2017, Crisan et al, Nature Communications, 2015 and Crisan et al, Stem Cell Reports, 2016.

Animals and other organisms

Policy information about studies involving animals; ARRIVE guidelines recommended for reporting animal research

Laboratory animals

All mice used in this study were males and females mixed between 3 and 6 months old except for embryos where the developmental stage was given in the document. C57BL/6J (Ly5.2) WT mice and Ly5.1 homozygous and heterozygous inbred mice were provided by our animal facilities. Mice were bred and housed at the Centre for Regenerative Medicine, Edinburgh, UK under a 12-hour light/12-hour dark cycle mimicking circadian rhythm and fed a chow diet and water ad libitum.
l. NG2Cre (B6;FVB-lfi208Tg(Cspg4-cre)1Akik/J) (Jackson Laboratories, stock #008533)
2. Runxlfl/fl (B6;129-Runxltm3.1Spe/J): Provided by Professor Nancy Speck (Available at Jackson Laboratories Stock# 010673)
3. Runxl-lRES-GFP: Provided by Prof James Downing
4. Rosa26-TdTomato mice (Gt(ROSA)26Sortm14(CAG-tdTomato)Hze) (Jackson Laboratories stock #007914)
5. C57BL/6J (Ly5.2) WT mice (CD45.2+CD45.1-) and Ly5.1 homozygous (CD45.2-CD45.1+) and heterozygous (CD45.2+CD45.1+) inbred mice were provided by our animal facilities.

Wild animals

This study did not involve wild animals.

Field-collected samples

This study did not involve field-collected samples.

Ethics oversight

All experiments were performed under a Project License granted by the Home Office (UK), approved by the University of Edinburgh Ethical Review Committee and conducted in accordance to local guidelines.

Note that full information on the approval of the study protocol must also be provided in the manuscript.

Flow Cytometry

Plots

Confirm that:

☒ The axis labels state the marker and fluorochrome used (e.g. CD4-FITC).

☒ The axis scales are clearly visible. Include numbers along axes only for bottom left plot of group (a 'group' is an analysis of identical markers).

☒ All plots are contour plots with outliers or pseudocolor plots.

☒ A numerical value for number of cells or percentage (with statistics) is provided.

Methodology

Sample preparation

AGMs were dissected and dissociated into single cells with collagenase type I (Sigma, C0130, 0.12% v/v). Fetal Liver cells were mechanically dissociated using a pipette and BM cells were flushed from both femurs and tibiae followed by red cell lysis with ammonium chloride solution (Stem Cell Technologies 07850) for 12 min at room temperature.

Instrument

BD LSR FortessaTM SORP Flow Cytometer, serial number H4D200001; Acea NovocyteTM 3000 Flow Cytometer, serial number 451140610822; BD Fusion Cell Sorter, serial number P658282F1001

Software

Data were collected using either BD FACS Diva Software V8.0.1 or NovoExpressTM Software.

Cell population abundance

The average percentage of analyzed/sorted cells, are given in Figures 1d, 7b, 7d, 7g, S2d, S2d and e, S3b.

Gating strategy

Cells were gated based on SSC-A vs. FSC-A, single cells were based on FSC-H vs. FSC-A, and live cells were gated based on the absence of a live/dead cell marker. Specific gates for different populations were determined using FMO controls.

☒ Tick this box to confirm that a figure exemplifying the gating strategy is provided in the Supplementary Information.
